# Pomalidomide enhances CD8^+^ T and NK cell mediated killing of HIV-infected cells

**DOI:** 10.1016/j.ebiom.2025.106004

**Published:** 2025-11-12

**Authors:** Rachel D. Pascoe, Celine Gubser, J. Judy Chang, Jan Schröder, Daniel T. Utzschneider, Alexander Barrow, Wen Shi Lee, James H. McMahon, Chris Y. Chiu, Ajantha Solomon, Jenny Anderson, Sharon R. Lewin, Thomas A. Rasmussen

**Affiliations:** a Department of Microbiology and Immunology, The University of Melbourne, at the Peter Doherty Institute for Infection and Immunity, Melbourne, Victoria, 3000, Australia; b Department of Infectious Diseases, The University of Melbourne, at the Peter Doherty Institute for Infection and Immunity, Melbourne, Victoria, 3000, Australia; c Department of Infectious Diseases, Alfred Hospital and Monash University, Melbourne, Victoria, 3004, Australia; d Victorian Infectious Diseases Service, Royal Melbourne Hospital at the Peter Doherty Institute for Infection and Immunity, Melbourne, Victoria, 3000, Australia; e Department of Infectious Diseases, Aarhus University Hospital, Aarhus, Denmark; f Department of Clinical Medicine, Aarhus University, Aarhus, Denmark

**Keywords:** Human immunodeficiency virus (HIV), CD8^+^ T-cells, NK cells, Immunomodulation, Exhaustion

## Abstract

**Background:**

Overcoming persisting immune dysfunction in people living with HIV (PLHIV) on suppressive antiretroviral therapy (ART) is a key challenge to curing HIV. Agents that reverse immune dysfunction may enhance viral immunity to support the immunological control and clearance of persisting HIV as part of curative strategies. Here, we investigated pomalidomide, a well-tolerated immunomodulatory drug, as an agent to enhance HIV-directed immune responses.

**Methods:**

We collected peripheral blood mononuclear cells (PBMC) from PLHIV on ART, cultured cells in the presence of HIV peptides and pomalidomide or DMSO control, and profiled the proliferative and cytotoxic functionality, and transcriptional landscape of HIV-specific CD8^+^ T-cells using MHC-I:HIV-peptide tetramers. *Ex vivo* pomalidomide-treated NK cells from PLHIV were also immunophenotyped, and assessed for polyfunctionality, ligand-mediated cytotoxicity and antibody-dependent cellular cytotoxicity (ADCC) against HIV-expressing target cells.

**Findings:**

Our findings demonstrated that pomalidomide significantly expanded tetramer-positive HIV-specific CD8^+^ T-cells with reduced markers of exhaustion, resulting in enhanced CD8^+^ T-cell-mediated killing of HIV-target cells. The expansion of CD8^+^ T-cells was associated with an upregulation of carbon metabolism and cell cycle pathways, with MYB and BATF3 key regulators of the pomalidomide-mediated response. Pomalidomide also enhanced NK cell killing of the human erythroleukemia K562 cell line and HIV-infected target cells by expanding polyfunctional cytotoxic NK cells with reduced TIGIT expression.

**Interpretation:**

Given the immune-enhancing effects and excellent safety profile, pomalidomide should be further investigated as an immune-enhancing strategy for an HIV cure.

**Funding:**

This study was supported by grants from the 10.13039/501100000925National Health and Medical Research Council of Australia (NHMRC), The Australian Centre for HIV and Hepatitis Research (ACH4), the Melbourne HIV Cure Consortium, J & M Wright Foundation, Australia, and the Independent Research Fund, Denmark.

**Research in context**

**Evidence before this study**

Antiretroviral therapy (ART) is unable to cure HIV due to the persistence of virus in a latent form in long-lived CD4^+^ T-cells. Furthermore, in people living with HIV (PLHIV), the chronic infection is associated with immune exhaustion, which impairs the clearance of latently infected cells and immunological control of HIV. There is therefore great interest in immune-modifying drugs that can enhance anti-HIV immunity and facilitate the elimination of infected cells. Pomalidomide, a licensed immunomodulatory agent, has previously demonstrated the capacity to improve NK cell and CD8^+^ T-cell functionality to clear malignancies, and supported the recall of HIV-specific T-cell responses in samples from PLHIV.

**Added value of this study**

Our study investigated pomalidomide and its capacity to enhance anti-HIV immunity and induce killing of HIV-infected cells. We found that pomalidomide reduced the exhaustion of HIV-specific CD8^+^ T-cells, reinvigorated HIV-specific CD8^+^ T-cell proliferative capacity and improved CD8^+^ T-cell clearance of HIV-target cells. The transcriptional regulators, MYB and BATF3, were associated with the increased proliferative capacity folloiwng pomalidomide treatment. Pomalidomide also reversed NK cell dysfunction in samples from PLHIV, improving NK cell cytotoxicity and killing of HIV-target cells.

**Implications of all the available evidence**

These data demonstrate that pomalidomide can enhance NK cell and HIV-specific CD8^+^ T-cell function and killing capacity *ex vivo*, providing pre-clinical data to further investigate pomalidomide, alone or with other agents *in vivo*, as a therapeutic strategy aimed at achieving durable control of HIV in the absence of ART.

## Introduction

Human immunodeficiency virus-1 (HIV) establishes a chronic infection that requires life-long antiretroviral therapy (ART) due to the persistence of long-lived and proliferating infected CD4^+^ T-cells.^1^ This viral reservoir contains replication-competent HIV that, in the absence of ART, results in viral rebound within 2–3 weeks.^2^ Chronic HIV infection leads to immune exhaustion and dysfunction of key immune effector cells that does not normalise on ART,^3^, ^4^, ^5^ with HIV-specific CD8^+^ T-cells typically displaying elevated expression of immune checkpoint (IC) markers, particularly programmed death-1 (PD-1) and T-cell immunoreceptor with Ig and ITIM domains (TIGIT),^6^^,^^7^ and limited proliferative capacity,^3^^,^^8^ indicative of a state of T-cell exhaustion. Exhaustion of Natural killer (NK) cells likely also contributes to persisting HIV infection, with CD56^−^CD16^+^ “dysfunctional” NK cells, characterised by weak cytolytic properties, greater exhaustion and senescence, expanded in PLHIV on ART.^9^^,^^10^ Given this, a key component of cure strategies is to re-invigorate dysfunctional HIV-specific immune responses to eliminate latently infected and/or virus-expressing cells that are responsible for viral rebound.^11^

Pomalidomide and lenalidomide are immunomodulatory imide drugs (IMiDs), both derivatives of thalidomide.^12^ Pomalidomide is licenced for the treatment of multiple myeloma and Kaposi Sarcoma, and is used in preference to thalidomide and lenalidomide due to better tolerance and potency.^13^^,^^14^ The primary target of pomalidomide is cereblon, a highly conserved E3 ubiquitin ligase.^15^^,^^16^ When pomalidomide and cereblon interact, cereblon ubiquitinates the IL-2-repressing zinc finger transcription factors, Ikaros and Aiolos, for degradation, leading to increased production of IL-2,^14^^,^^17^ supporting immune function.^18^

The enhanced immune function by pomalidomide has been implicated in improved clinical outcomes in some cancers, with pomalidomide treatment associated with greater polyfunctional T-cells, and interferon-γ (IFNγ) and tumour necrosis factor (TNF)-α-producing NK cells in lenalidomide-refractory relapsed myeloma.^18^ In PLHIV, administration of pomalidomide at 5 mg for 21 days for the treatment of Kaposi Sarcoma activated CD4^+^ and CD8^+^ T-cells, reduced T-cell senescence and expanded central memory CD4^+^ and CD8^+^ T-cells.^19^ *In vitro*, pomalidomide enhanced HIV-specific CD4^+^ and CD8^+^ T-cell responses, characterised by an increase in IFNγ and TNFα-producing cells following stimulation with HIV Gag or Nef peptides.^20^ Pomalidomide also enhanced T-cell responses against Human T-cell Leukaemia Virus Type I (HTLV-I) in HTLV-1 infected macaques.^21^

Pomalidomide has also been implicated in HIV latency reversal; however, our investigations found no effect of pomalidomide on HIV latency reversal, susceptibility of the reservoir to immune-mediated killing, nor induction of apoptosis.^22^ Given this, we propose that the primary benefit of pomalidomide in the setting of HIV infection would be through its immunomodulatory effects, and hypothesised that pomalidomide would enhance anti-HIV immunity, increasing the clearance of HIV-infected cells and could potentially be used as a component of an HIV cure strategy.

## Methods

### Experimental model and subject details

PBMC from HIV-negative donors were isolated by Ficoll–Paque density gradient centrifugation (Cytiva) from buffy coats obtained from the Australian Red Cross Lifeblood (Melbourne, Australia). PBMC were treated with ACK lysing buffer (Gibco, A10492-01) to remove erythrocytes, and rested for 24 h at 2 × 10^6^ cells/mL before culturing at 1 × 10^6^ cells/mL in assay conditions. PBMC from PLHIV were obtained by leukapheresis at the Alfred Hospital (Melbourne, Australia), isolated by Ficoll–Paque density gradient centrifugation, resuspended in FBS with 10% DMSO and stored in liquid nitrogen. Upon thawing, PBMC were rested overnight in the presence of HIV integrase inhibitor raltegravir (1 μM; Selleck Chem). PBMC were then cultured with the appropriate drug conditions at 1-1.5 × 10^6^ cells/mL in the presence of ART (raltegravir). All study participants were male. Plasma HIV RNA was below the detection limit of the utilised assay (<20 or <40 copies/mL) at the time of leukapheresis. The mean duration of suppressed plasma HIV RNA was 14.8 years (95% confidence interval (CI) 10.69–18.91), and the mean nadir was 306.2 cells/μL (95% CI 188.5–424.0). Additional patient demographics and ART regimen listed in Table S1.

### Cell lines

K562 (obtained from A. Barrow, The University of Melbourne, Australia), and LAV/8E5 (NIH; ARP-095) cell lines were cultured in RPMI 1640 medium (Life Technologies) supplemented with 10% (v/v) heat inactivated foetal bovine serum (FBS), 100 U/mL penicillin, 100 μg/mL streptomycin, and 292 μg/mL glutamine (Gibco) at 37 °C and 5% CO_2_. HEK 293T (obtained from NIH), and TZM-bl (NIH; HRP-8129) cell lines were grown as monolayer in Dulbecco's minimal essential medium (DMEM, Gibco) supplemented with 10% (v/v) heat inactivated foetal bovine serum (FBS), 100 U/mL penicillin, 100 μg/mL streptomycin, and 292 μg/mL glutamine (Gibco) at 37 °C and 5% CO_2_. Validation of cell lines can be found in Table S2. Cell lines were commercially sourced and free of mycoplasma.

### Cell culturing conditions

Pomalidomide (Sigma Aldrich; P0018) was assessed in PBMC cultures of uninfected donors at the therapeutically relevant concentration of 0.25 μM, which corresponds to the C_MAX_ of *in vivo* pomalidomide administration,^23^ compared to the DMSO vehicle control. The DMSO input was normalised to that of pomalidomide at 0.06%. Pomalidomide was tested independently or in combination with T-cell antigenic stimulant staphylococcal enterotoxin B (SEB; 0.125 ng/mL; Sigma Aldrich). The concentration of SEB required to induce low-level stimulation through TCR-MHC class II (MHC–II)–specific interactions was determined by titration in a culture of PBMC, measuring CD4^+^ T-cell and CD8^+^ T-cell activation. For co-stimulation of PBMC from HIV-positive donors, Gag peptide pool (NIH; 8117), Nef peptide pool (NIH; 12425), and cytomegalovirus, Epstein–Barr virus, and influenza virus (CEF) peptide pool (StemCell Technologies; 100-0675) were used at 0.5 μg/mL. The Gag peptide pool comprised 123 overlapping peptides of 15- or 12-mers, with 11 amino acid overlaps, and spanning the entire HIV-1 subtype B Gag region. The Nef peptide pool comprised 49 overlapping peptides of predominantly 15-mers with 11 amino acid overlaps spanning the entire HIV-1 subtype B Nef region. The CEF peptide pool consisted of 32 peptides of defined HLA-I restricted T-cell epitopes of the three viruses. 100 IU/mL of IL-2 recombinant human protein (R&D Systems; RDS202IL500) was used for IL-2 comparative assays.

### *Ex vivo* phenotyping studies of PBMC from people living with HIV

Phenotypes of PBMC from PLHIV treated *ex vivo* with pomalidomide (n = 12) were assessed in a large flow panel using the antibodies described in Table S3, as previously described.^22^ All antibodies used in this study were commercially sourced, and titrated and validated by single staining and complete panel staining in multiple donors. Briefly, cells were stained with Zombie NIR Fixable Viability Kit (Biolegend; 423106) for 15 min at room temperature, then stained with the antibody cocktail for 25 min at room temperature. Cells were fixed with 1% paraformaldehyde, and acquisitions were performed on the Cytek Aurora. Flow cytometry analyses were conducted using OMIQ software.

### Peptides and peptide-HLA class I tetramers

HIV peptides shown to bind to HLA-A∗02, HLA-A∗03, HLA-B∗07, and HLA-B∗08 (A2-SL9/N_77-85_ SLYNTVATL; A2-IV9/N_309-317_ ILKEPVHGV; A3-RK9/N_20-28_ RLRPGGKK; B8-EI8/N_128-135_ EIYKRWII) were ordered from the NIH AIDS Reagent program, and Genscript. Tetramers were generated and validated as described.^24^^,^^25^

### HLA typing

HLA typing, measuring HLA Class I alleles (HLA-A,-B, -C) and HLA Class II alleles (HLA-DRB, -DQ, -DPB1), was performed on our ART-suppressed PLHIV PBMC repository using Illumina MiSeq next-generation sequencing at the Institute for Immunology and Infectious Diseases, Murdoch University, Perth, Australia.

### HIV-specific CD8^+^ T-cell surface immunophenotyping

PBMC from HLA-typed PLHIV (n = 7) were thawed, and one-third PBMC were pulsed with an immunodominant HIV peptide (1 μg/L) for 1 h, and then added back to whole PBMC and placed in culture for 13 days with pomalidomide (0.25 μM) or DMSO, in the presence of ART. On day 4, media was changed, with the addition of 10 U/mL IL-2, and media changes were continued every 2 days. On day 13, cells were harvested from the peptide-stimulated cultures. To phenotype HIV-specific CD8^+^ T-cells, PE-conjugated HLA-matched tetramers corresponding to the peptide stimulation were used and cells were stained for surface markers as described,^24^ with the inclusion of anti-TIGIT-BV605 (A15153G; Biolegend). Flow cytometry analyses were conducted using OMIQ software.

### HIV-specific CD8^+^ T-cell proliferation assay

For proliferation assays, proliferation labelling was performed using CellTrace Violet Proliferation Stain. PBMC (n = 7) were cultured as described above for HIV-specific CD8^+^ T-cell analysis, with one-third of PBMC pulsed with an immunodominant peptide, and cultured in the presence of pomalidomide or DMSO for 13 days in ART-containing media, with frequent media changes. Staining was performed at baseline, and on days 4, 7, 10 and 13. Cells were stained with Live/Dead Fixable-eFluor780 Dead Cell Stain (Life Technologies; 65-0865-14) with Fc block, washed and then stained with the PE-conjugated HLA-matched tetramers corresponding to the peptide stimulation. Surface staining was performed with the antibodies described in Table S4.

### HIV-specific CD8^+^ T-cell killing assay

CD8^+^ T-cells were sorted on day 13 from pomalidomide or DMSO-cultured PBMC following exposure to an immunodominant HIV peptide (n = 7). The HIV-specific CD8^+^ T-cell killing assay was performed as previously described.^24^^,^^25^ Target CD4^+^ T-cells were thawed, sorted and rested overnight prior to staining and co-culturing. CD8^+^ T-cell input was normalised based on the frequency of tetramer^+^ HIV-specific CD8^+^ T-cells for a 2:1:1 (Tetramer^+^ CD8^+^ T-cell: peptide-loaded CD4^+^ T-cell: non-peptide loaded CD4^+^ T-cell) for 6 h. Total CD8^+^ T-cells were also cultured with CD4^+^ T-cells, cultured 4:1:1, 2:1:1, and 1:1:1 (Total CD8^+^ T-cell: peptide-loaded CD4^+^ T-cell: non-peptide loaded CD4^+^ T-cell). Co-cultures were performed in the absence of DMSO or pomalidomide.

### RNA extraction, sequencing and analysis of HIV-specific CD8^+^ T-cells

CD8^+^ T-cells were sorted on day 13 from pomalidomide or DMSO-cultured PBMC following exposure to an immunodominant HIV peptide (n = 6). HIV-specific CD8^+^ T-cells were stained as described in the HIV-specific CD8^+^ T-cell Killing assay and Live^+^CD3^+^CD8^+^Tetramer^+^ cells were sorted on the BD FACSAria Fusion Cell Sorter, with RNA extracted using the Qiagen RNeasy Plus Micro Kit (Cat no. 74034), per the manufacturer's instructions. mRNA quality was confirmed using TapeStation (4200 TapeStation System, Agilent Technologies). RNA-seq libraries were prepared from 100 ng of RNA using the TruSeq RNA Library Prep Kit (Illumina) according to the manufacturer's instructions. Sequencing was performed on the Illumina NextSeq 500 instrument generating 132 base reads, using the Illumina P2 100cycle kit (Illumina).

Sequencing reads were aligned to the GRCh38 reference genome and transcriptome (version 105) using the STAR aligner (version 2.7.8a),^26^^,^^27^ and transcript counts were established using featureCounts from the subread package (version 2.0.0).^28^ The data was then analysed using R (version 4.2.0), edgeR (version 3.40.2)^29^ and plots generated with ggplot2.^30^ To identify differentially expressed genes between the experimental groups, we first filtered the data for the “No treatment” condition and lowly or non-expressed genes (filterByExpr function) and normalised counts by TMM. From the initial MDS plots, we identified a batch effect between the donors, which we used as covariates in the design matrix to regress the batch effect. We estimated group-wise dispersion with the estimateDisp (robust = TRUE) function and fit a linear model using glmFit. We tested for differential expression between the Pom and DMSO groups using the glmLRT function.

We used the barcodeplot function to display gene set enrichment and ROAST to statistically test the significance. The heatmap of expression values of differential core exhaustion genes was generated with the pheatmap package (version 1.0.12) on log-normalised CPM values regressed for batch effect. To analyse potential regulating transcription factors, we used the RTN package.^31^ We ran 1000 permutations of network analysis on 961 regulons, followed by standard bootstrapping and filtering steps. Top regulators were identified by intersecting the regulons with differentially expressed genes and running transcriptional network analysis (TNA). Association plots were generated using the tna.gsea2 function.

The protein–protein interaction (PPI) network was constructed using Cytoscape^32^ and the STRING Database. The top 200 differentially expressed genes (DEG) were evaluated in the STRING database, with 50 genes highlighted in the PPI network as genes of interest.

### HIV-specific CD8^+^ T-cell intracellular staining

To measure HIV-specific CD8^+^ T-cell responses, on day 13, isolated CD8^+^ T-cells were re-stimulated with the immunodominant peptide (2 μg/mL) for 6 h with Golgiplug (BD; 555029) and Golgistop (BD; 554724), and anti-CD107a-BUV395 (H4A3; BD) (n = 7). Tetramer staining was performed, as described above, with Live/Dead-Violet and Fc block, and surface staining was performed with the antibodies described in Table S5. Intracellular cytotoxic molecules were measured with the antibodies described in Table S5 using the eBioscience Foxp3/Transcription Factor Staining Buffer Set and BD Perm Wash solution (BD; 544714). Cells were fixed with 1% paraformaldehyde, and acquisitions were performed on the BD LSRFortessa. Flow cytometry analyses were conducted using OMIQ software.

### K562 assay

NK cell cytotoxicity following pomalidomide treatment was assessed in a standard assay against K562 target cells (NIH) with 40:1, 20:1, 10:1, and 1:1 E:T ratio, as described.^33^ Briefly, the NK target cell line, K562 was stained using Cell Proliferation Dye eFluor670 (Invitrogen, 65-0840-90), as per the manufacturer's recommendations. Stained K562 cells were co-cultured with PBMC from PLHIV (n = 9) that had been thawed and rested overnight, in the presence of various pomalidomide drug conditions for 48 h. Cells were harvested and stained with Live/Dead Fixable-Violet Dead Cell Stain to quantify viability in eFluor670-stained K562 target cells. Direct cytotoxicity by NK cells was measured as a change of viable K562 cells relative to K562 cells in the absence of effector cells. Cells were fixed with 1% paraformaldehyde, and acquisitions were performed on the BD LSRFortessa. Flow cytometry analyses were conducted using Flowjo.

### Virus and infection

Full-length, Nef-competent EGFP-reporter HIV was produced as previously described.^34^ Virus supernatant was harvested 2 days after transfection, filtered by passage through 0.45 μM filter and concentrated using Lenti-X Concentrator (Takarabio; 631232), per manufacturer's instructions, and stored in aliquots in −80 °C. TCID50 was determined on TZM-bl cells using the Reed and Muench method,^35^ and read using luciferase as a read-out, as described previously^36^ (Promega; E1501).

### Autologous NK cell HIV killing assay

Total CD4^+^ T-cells (≥96 purity assessed using Live/Dead Fixable-Violet Dead Cell Stain (Invitrogen; L34955), anti-CD3-PE (HIT3a; BD) and anti-CD4-FITC (RPA-T4; BD)) were negatively selected using magnetic cell sorting with anti-human CD4^+^ T-cell Isolation Kit (Miltenyi Biotech; 130-096-533) (n = 7). CD4^+^ T-cells were stimulated with PHA (10 μg/mL) and IL-2 (10 U/mL) for 48 h. Simultaneously, PBMC were rested for 24 h before being incubated with pomalidomide or DMSO, in the presence of low-level SEB, as described above. On day 3, stimulated CD4^+^ T-cells were infected with full-length, Nef-competent EGFP-reporter virus at TCID50 = 1 for 2–3 h, washed and continued in culture with IL-2 (10 U/mL) for 48 h. On day 5, NK cells were purified via negative selection (Miltenyi Biotech, 130-092-657) from treated PBMC. Simultaneously, infected CD4^+^ T-cells were stained with Cell Proliferation Dye eFluor670. CD4^+^ T-cells and NK cells were co-cultured at various E:T ratios of 1:1, 1:2, 1:4, 1:10 or CD4^+^ T-cells alone in 96-well plates in the absence of DMSO or pomalidomide, but in the presence of raltegravir. Cultures were harvested at 18 h and stained with Live/Dead Fixable-Violet Dead Cell Stain to quantify changes in the frequency of viable GFP-expressing HIV-infected CD4^+^ T-cells. Cells were fixed with 1% paraformaldehyde, and acquisitions were performed on the BD LSRFortessa. Flow cytometry analyses were conducted using Flowjo. The percentage of killing (%killing) of HIV-infected CD4^+^ T-cells by NK cells was calculated using the following formula: *[(%GFP*^*+*^ *cells in targets) − (%GFP*^*+*^ *cells in targets + effectors)]/(%GFP*^*+*^ *cells in targets) × 100.*

### Flow cytometry-based infected cell elimination (ICE) assay

To assess the effect of pomalidomide on ADCC, *ex vivo*-treated NK cells from PLHIV (n = 7) were assessed for ADCC against the target HIV cell line, LAV/8E5, mediated by HIV-1^+^ sera, as previously described with minor modifications.^37^ PBMC from PLHIV were thawed and rested overnight ahead of treatment with pomalidomide or DMSO for 72 h. NK cells were negatively selected using EasySep Human NK Cell Isolation Kit (StemCell Technologies, 17955). NK cells were co-cultured with eFluor670-stained HIV-1 target cell line, LAV/8E5 (NIH AIDS Reagent Program) at 1:1 ratio, in the presence of polyclonal HIVIG (NIH AIDS Reagent Program), or IgG from human serum (Sigma Aldrich, I4506) at 50 μg/mL for 4 h. Co-cultures were performed in the absence of DMSO or pomalidomide. Post-incubation, the target cells were stained for viability using Live/Dead Fixable-Violet Dead Cell Stain as described above. Intracellular p24 was measured using BD Cytofix/Cytoperm 1× solution (BD; 544714) for 20 min on ice, then stained with anti-KC57-RD1 p24-PE (Beckman Coulter; 6604667). The percentage of killing (%killing) of target cells by NK cells was calculated using the following formula: *[(%p24*^*+*^ *cells in targets) − (%p24*^*+*^ *cells in targets + effectors ± HIV-1*^*+*^ *serum)]/(%p24*^*+*^ *cells in targets) × 100, as described*.^37^ Cells were fixed with 1% paraformaldehyde, and acquisitions were performed on the BD LSRFortessa. Flow cytometry analyses were conducted using Flowjo.

### NK cell intracellular cytokine staining

PBMC from PLHIV (n = 8) were thawed, rested overnight, and pre-treated in the presence of pomalidomide or DMSO for 24, 48, and 72 h in the presence of ART. PBMC were then resuspended for 6 h with brefeldin A (5 μg/mL; Sigma Aldrich; B7651-5 MG), monensin (5 μg/mL; Sigma Aldrich; M5273) and anti-CD107a-AF647 (H4A3; BD). In the last 4 h of culture, PBMC were exposed to target K562 cells to induce NK responses. NK cell responses were then measured using the antibodies described in Table S6. Briefly, cells were stained with LiveDead Fixable Blue Viability Stain (Invitrogen; L23105) for 15 min at room temperature, then stained with the surface antibody cocktail for 25 min at room temperature. Cells were then washed and permeabilised using BD Cytofix/Cytoperm (BD; 544714) prior to staining with the intracellular antibody cocktail for 1 h. Acquisitions were performed on the Cytek Aurora, and flow cytometry analyses were conducted using OMIQ software.

### Study ethics approval

Large numbers of peripheral blood mononuclear cells (PBMCs) were collected from PLHIV on suppressive ART by leukapheresis at the Alfred Hospital, Melbourne, Australia, after obtaining written informed consent with approval for their use for research from the relevant Institutional Review Boards (Alfred Hospital, Melbourne, Australia, HREC 214/15 & 48/16; The Avenue Hospital, Windsor, Australia, HREC 00242, protocol 202; The University of Melbourne, Australia, HREC 15452271 & 1443071). Inclusion criteria were being HIV antibody positive and on suppressive ART with viral load <50 copies/ml for at least 3 years. Participants were also negative for HBsAg, HCV antibodies and HCV RNA. Buffy coats were obtained from the Australian Red Cross Lifeblood (Melbourne, Australia) with approval from the relevant Institutional Review Boards (The University of Melbourne, Australia, HREC 1443071).

### Statistics

FlowJo, OMIQ, GraphPad Prism, Pestle and SPICE software were used for analysis. Data was examined for normal distribution using GraphPad Prism's normality algorithm, and visual inspection of frequency histograms and quantile–quantile plots. Parametric or non-parametric tests were then applied as appropriate, with statistical comparisons between experimental conditions performed by signed-rank tests. Correlations between measured cell parameters were explored using Spearman's or Pearson's correlation. Synergy was determined using the Bliss Independence Model. Illustrations were generated with Biorender.com.

### Role of funders

This study was funded by grants from the National Health and Medical Research Council of Australia (NHMRC; program grant and practitioner fellowship), The Australian Centre for HIV and Hepatitis Research (ACH4), the Melbourne HIV Cure Consortium, the J & M Wright Foundation, Australia, and the Independent Research Fund, Denmark. Funders did not have any role in the study design, data collection, data analyses, interpretation, or the writing of the report.

## Results

### Pomalidomide activates CD8^+^ T-cells and synergises with antigen stimulation

HIV-negative donors and PLHIV on suppressive ART for at least three years were recruited to the study. The clinical characteristics of PLHIV are shown in Table S1. Peripheral blood mononuclear cells (PBMC) were isolated from donors, and the effects of pomalidomide were assessed *ex vivo* on CD8^+^ T-cell differentiation, activation, exhaustion, and senescence. We evaluated two concentrations of pomalidomide in samples from ART-suppressed PLHIV: a commonly used concentration of 2 μM described in *in vitro* cancer investigations,^14^^,^^15^^,^^38^ and a clinically relevant concentration of 0.25 μM, which is consistent with the maximal plasma concentration (C_MAX_) in HIV-negative individuals and PLHIV who received 5 mg pomalidomide daily for Kaposi Sarcoma, which was well tolerated.^23^

**Fig. 1:**
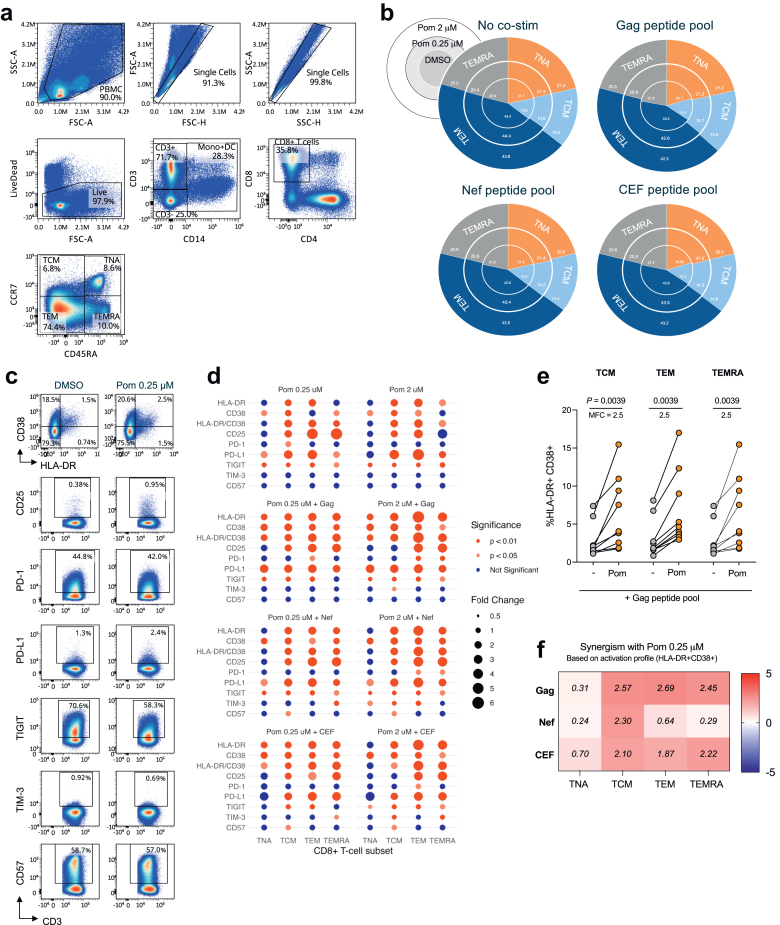
**Pomalidomide activates memory CD8^+^ T-cells, and synergises with antigen stimulation**. PBMC from PLHIV were treated with pomalidomide (0.25 μM or 2 μM) or DMSO, in the presence of Gag peptide pool, Nef peptide pool, CEF peptide pool, or no co-stimulation for 72 h *ex vivo*. (**a**) Representative flow plots of the gating strategy for CD8^+^ T-cell subsets; T_NA_ = naïve, T_CM_ = Central memory, T_EM_ = effector memory, T_EMRA_ = CD45RA^+^ effector memory. (**b**) Pie charts represent the median subset contribution to the CD8^+^ T-cell population (n = 9). (**c**) Representative FACS plots of key surface markers: HLA-DR, CD38, CD25, PD-1, PD-L1, TIGIT, TIM-3, CD57. (**d**) Dot plot of the expression of measured surface markers within CD8^+^ T-cell subsets following pomalidomide treatment, relative to the DMSO condition, with the respective co-stimulatory peptide pool. The size of the dot reflects the relative fold change, and the colour indicates the significance of the expression change. (**e**) Frequency of HLA-DR^+^CD38^+^expressing cells in CD8^+^ T-cell memory subsets following treatment with pomalidomide (0.25 μM) or DMSO, in the presence of Gag peptide pool. (**f**) Heatmap of the median scores from the Bliss Independence Model, testing for synergy between pomalidomide (0.25 μM) and the various antigen co-stimulation agents. The difference in the activation (HLA-DR+ CD38+) of CD8^+^ T-cell subsets from DMSO control was used as the comparative metric. Positive scores (greater than zero) indicate synergy, negative scores (less than zero) indicate antagonism, and scores of zero indicate an additive effect. MFC = mean fold change. (n = 9; Wilcoxon matched-pairs signed rank test).

To determine whether antigenic co-stimulation might augment the effects of pomalidomide, we tested pomalidomide in the absence of any antigen co-stimulation, or in the presence of overlapping peptides to HIV antigens (Gag or Nef), or cytomegalovirus, Epstein–Barr virus, and influenza virus (CEF) antigens for 72 h. Compared to DMSO vehicle control, pomalidomide did not change the distribution of CD8^+^ T-cell subsets (Fig. 1a and b) but strongly induced CD8^+^ T-cell activation with significant increases in HLA-DR, CD38 and CD25 (Fig. 1c and d), and 2.5-fold increases in the co-expression of HLA-DR and CD38 within all CD8^+^ T-cell memory subsets (p = 0.0039, p = 0.0039, and p = 0.0039, respectively; Fig. 1e), consistent with previously published observations of pomalidomide activating T-cells.^39^^,^^40^ Both concentrations of pomalidomide tested had similar stimulatory effects and minimal impact on the expression of immune checkpoints, with the exception of a consistent elevation of PD-L1 and downregulation of the inhibitory receptor, TIGIT, in naïve (T_N_), central memory (T_CM_) and effector memory (T_EM_) CD8^+^ T-cells, seen with both concentrations of pomalidomide (Fig. 1d). Notably, the pomalidomide stimulatory effects were most pronounced in the presence of antigen, as observed with both of the examined HIV peptide pools (Gag and Nef), and the CEF peptide pool, inducing stronger activation of memory CD8^+^ T-cell subsets than DMSO (Fig. 1f). Taken together, these analyses show that pomalidomide promotes CD8^+^ T-cell activation and its activity is greater with antigen co-stimulation, without inducing markers of T-cell exhaustion.

### Pomalidomide drives expansion of HIV-specific CD8^+^ T-cells with reduced PD-1 expression

**Fig. 2:**
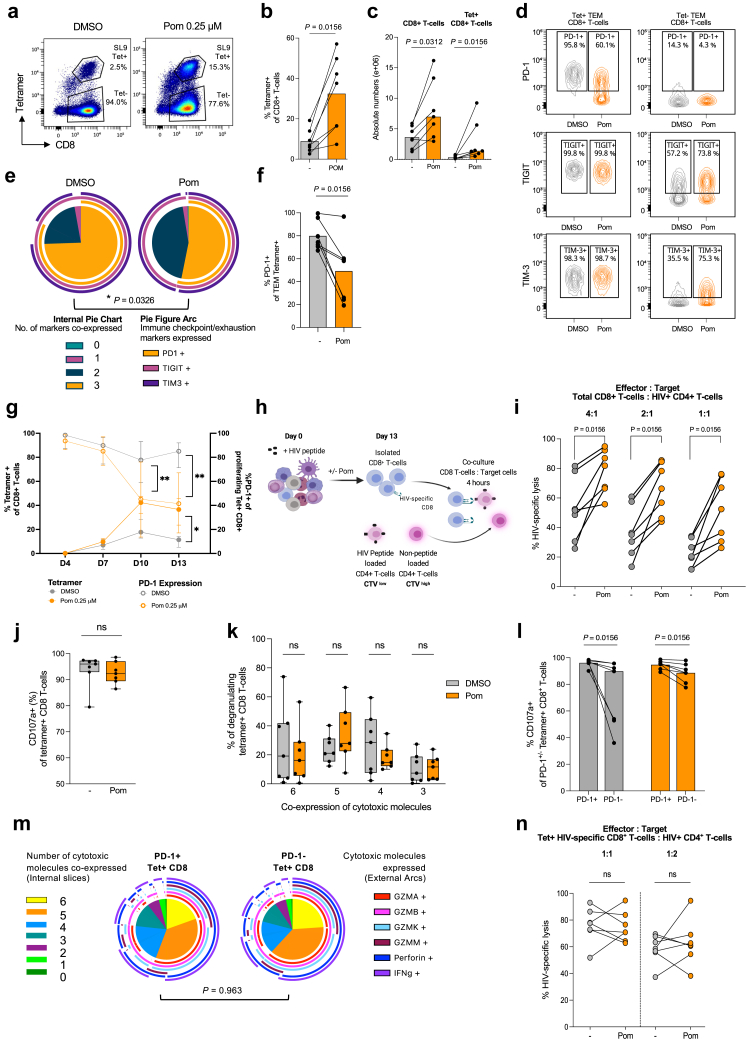
**Pomalidomide reduces PD-1 expression, supporting greater expansion of HIV-specific CD8^+^ T-cells to enhance killing of HIV+ CD4+ T-cells**. HIV-specific CD8^+^ T-cell responses were measured after 13 days culturing of PBMC from ART-suppressed PLHIV with DMSO or pomalidomide (0.25 μM), in the presence of an immunodominant HIV peptide. HIV-specific CD8^+^ T-cells were measured using a tetramer to the same immunodominant HIV peptide. (**a**) Representative flow plots of tetramer staining for HIV-specific CD8^+^ T-cells (pre-gated on live CD3^+^). (**b**) Frequency of tetramer^+^ HIV-specific CD8^+^ T-cells of the CD8^+^ T-cell pool. DMSO-treated conditions indicated in grey, pomalidomide-treated conditions indicated in orange. (**c**) Absolute number of CD8^+^ T-cells, and tetramer^+^ HIV-specific CD8^+^ T-cells after 13 days of treatment, following equal numbers cultured at baseline. (**d**) Representative staggered overlayed contour plots showing the gating of PD-1, TIGIT and TIM-3 in DMSO-treated and pomalidomide-treated tetramer^+^ and tetramer-effector memory (T_EM_) HIV-specific CD8^+^ T-cells after the 13 days culture. (**e**) Pie-charts representing the average fractions of tetramer^+^ T_EM_ CD8^+^ T-cells co-expressing different immune checkpoints (IC) markers (internal slices), and their expression profiles (external arcs). Statistical significance (p < 0.05) was determined by permutation tests. (**f**) PD-1 expression in T_EM_ tetramer^+^ HIV-specific CD8^+^ T-cells. (**g**) PBMC from PLHIV were pre-stained with the proliferation dye, CTV, and treated with DMSO or pomalidomide (0.25 μM) for 13 days, following exposure to an immunodominant HIV peptide, as described. The frequency of tetramer+ HIV-specific CD8^+^ T-cells (full circles, left y-axis), and the expression of PD-1 within the proliferating HIV-specific CD8^+^ T-cell population (circle outline, right y-axis) was quantified on days 4, 7, 10, and 13. DMSO indicated in grey, and pomalidomide-treated indicated in orange. The median of 7 donors shown. (**h**) Schematic of CD8^+^ T-cell killing assay. PBMC from PLHIV were stimulated with a HIV-immunodominant peptide and treated with DMSO or pomalidomide (0.25 μM) for 13 days. Purified CD8^+^ T-cells were co-cultured with untreated autologous CD4^+^ T-cells stained with two CTV concentrations, with the lower concentration loaded with the HIV immunodominant peptide. HIV-specific lysis was calculated as the relative killing of the peptide-loaded CD4^+^ T-cells to the non-peptide-loaded CD4^+^ T-cells. (**i**) Percentage of HIV-specific lysis by DMSO and pomalidomide-treated CD8^+^ T-cells at various E:T ratios, with CD8^+^ T-cell effector input normalised to total CD8^+^ T-cell (E): peptide-loaded CD4^+^ T-cells (T). (**j**) Frequency of tetramer^+^ HIV-specific CD8^+^ T-cells degranulating (CD107a+) following HIV antigen restimulation with the same HIV peptide. (**k**) Frequency of tetramer+ CD8^+^ T-cells co-expressing 3-, 4-, 5-, or 6 cytotoxic molecules following peptide restimulation. (**l**) Frequency of degranulation (CD107a+) within PD-1-expressing (PD-1+) and PD-1-negative (PD-1-) tetramer+ HIV-specific CD8^+^ T-cells following HIV antigen restimulation. (**m**) Pie-charts representing the cytotoxic molecules profile of degranulating pomalidomide-treated PD-1 expressing and PD-1 negative tetramer^+^ HIV-specific CD8^+^ T-cells. Co-expression of different cytotoxic molecules (internal slices) and their expression profiles (external arcs) shown. (**n**) Percentage of HIV-specific lysis by DMSO and pomalidomide-treated CD8^+^ T-cells using the HIV CD8+ T-cell killing assay. Individual cytolytic capacity was measured by normalising effector input as tetramer^+^ HIV-specific CD8^+^ T-cell (E) to peptide-loaded CD4^+^ T-cells (T) at various effector(E):target(T) ratios. (n = 7; Wilcoxon matched-pairs signed rank test. ∗p < 0.05, ∗∗p < 0.01; ns, not significant. Bars show median+ IQR. Box plot whiskers show the min/max values. Fig. 2m statistical significance (*P* < 0.05) determined by permutation tests).

We next investigated whether pomalidomide, in addition to inducing CD8^+^ T-cell activation, would also expand HIV-specific CD8^+^ T-cells following exposure to an HIV immunodominant peptide. PBMCs from HLA-typed PLHIV on ART were pulsed with an immunodominant Gag or Pol peptide and cultured with pomalidomide or DMSO for 13 days. HIV-specific CD8^+^ T-cells were quantified using previously validated HIV-specific tetramers binding to either HLA-A2, -A3, or -B8 presenting the cognate Gag or Pol peptide^24^ (Fig. 2a, Figure S1a). The median baseline tetramer^+^ HIV-specific CD8^+^ T-cell frequency as a percentage of total CD8^+^ T-cells was 0.15% (range 0.06–0.76%). Pomalidomide treatment, compared to DMSO, led to a substantial expansion of HIV-specific CD8^+^ T-cells with a 4.01-fold-increase in the frequency of HIV-specific CD8^+^ T-cells (p = 0.0156; Fig. 2b) with no decrease in viability (Figure S1b). No correlation was found between baseline tetramer levels and the fold increase in tetramer+ cells by day 13 (Figure S1c). The absolute number of tetramer^+^ HIV-specific CD8^+^ T-cells increased by 9.26-fold following pomalidomide treatment (p = 0.0156), which contributed to the 2.35-fold increase in absolute numbers of CD8^+^ T-cells following pomalidomide treatment. The pomalidomide-induced expansion of tetramer+ CD8^+^ T-cells as a percentage of the live PBMC pool was substantially greater than that observed in the tetramer-negative CD8^+^ T-cell population, with a 4.1-mean fold change (p = 0.0312) and 2.6-mean fold change (p = 0.0156), respectively (Fig. 2c, Figure S1d).

Tetramer^+^ HIV-specific CD8^+^ T-cells were predominantly T_EM_, with a minor increase in the proportion of T_EM_ following pomalidomide treatment (Figure S1e). Within the tetramer^+^ CD8^+^ T_EM_ population, pomalidomide significantly altered the IC expression profile, with reduced co-expression of all three measured ICs: PD-1, TIGIT and TIM-3 (p = 0.0326; Fig. 2d and e), driven by a distinct reduction of PD-1 expression (p = 0.0156; Fig. 2f).

Reduced PD-1 expression was accompanied by improved proliferative capacity, with proliferating (CTV low) pomalidomide-treated HIV-specific CD8^+^ T-cells expressing significantly less PD-1 compared to DMSO (p = 0.0156; Fig. 2g, Figure S1f and g). The pomalidomide-induced downregulation of PD-1 was not limited to proliferating cells, with reduced PD-1 MFI also observed within the non-proliferating population (CTV high) (p = 0.0312; Figure S1h and i).

Pomalidomide treatment and exposure to an HIV-immunodominant peptide also changed the distribution of T-cell subsets within the tetramer- CD8^+^ T-cells, with greater frequency of T_EM_ CD8^+^ T-cells observed (p = 0.0156; Figure S1e). This was accompanied by an increased proportion of tetramer- CD8^+^ T-cells that underwent proliferation after 4 days exposure, albeit at a reduced rate than the tetramer^+^ HIV-specific CD8^+^ T-cells (Figure S1j). In the tetramer- CD8^+^ T-cells, pomalidomide induced a significant expansion of PD-1^+^TIGIT^+^TIM-3^+^ T_EM_ (p = 0.0469), which was mainly driven by an increased expression of TIM-3 (Figure S1k). Finally, we assessed the effects of pomalidomide on CD8- T-cells, which are predominantly CD4^+^ T-cells (>95%) and found no effect of pomalidomide on the proliferation of these cells (Figure S1j). In fact, pomalidomide treatment following exposure to an HIV immunodominant peptide impaired the proliferation of CD3+CD8- T-cells in 5/7 donors by 0.7-median fold.

### Pomalidomide expansion of HIV-specific CD8^+^ T-cells increases HIV-specific killing without enhancing per-cell cytotoxicity

Given the greater frequency of tetramer+ HIV-specific CD8^+^ T-cells with reduced PD-1 expression following pomalidomide treatment, we next tested the ability of pomalidomide to enhance CD8^+^ T-cell killing capacity by employing an HIV-specific CD8^+^ T-cell killing assay.^25^^,^^41^ HIV-peptide-recalled HIV-specific CD8^+^ T-cells were cultured with untreated autologous CD4^+^ T-cells loaded with the same HIV peptide at various effector-target (E:T) ratios (Fig. 2h). HIV-specific killing was measured by the relative loss of the peptide-loaded (CTV^low^) viable CD4^+^ T-cells relative to the non-peptide-loaded (CTV^high^) viable CD4^+^ T-cells. We observed a significant increase in HIV-specific CD8^+^ T-cell killing by the pomalidomide-treated CD8^+^ T-cell pool, with a 1.69-fold (p = 0.0039), 2.15-fold (p = 0.0039), and 2.53-fold (p = 0.0039) increase of killing at E:T ratios 4:1, 2:1, and 1:1, respectively (Fig. 2i). Taken together, this demonstrates that through expanding the pool of HIV-specific CD8^+^ T-cells, pomalidomide can induce HIV-specific killing.

This profile was accompanied by no alteration in the proportion of HIV-specific CD8^+^ T-cells that underwent degranulation (Fig. 2j), which were the majority of tetramer^+^ CD8^+^ T-cells. Quantification of the production of cytotoxic molecules, granzyme-A, B, K, M, perforin and IFNγ revealed an increase in granzyme A following pomalidomide treatment relative to DMSO (p = 0.0469), with a simultaneous reduction in granzyme K in degranulating tetramer^+^ HIV-specific CD8^+^ T-cells (p = 0.0312; Figure S2a). This produced a distinct change in the co-expression profiles of HIV-specific tetramer^+^ CD8^+^ T-cells (Figure S2b and c), but a similar median proportion of cells co-expressing all six cytotoxic molecules in DMSO- and pomalidomide-treated tetramer^+^ CD8^+^ T-cells (Fig. 2k).

Given the improved proliferative capacity associated with the reduced PD-1 expression on HIV-specific tetramer+ CD8^+^ T-cells, we next investigated whether a reduction in PD-1 expression also conferred improved cytotoxicity. We found a reduction in the expression of CD107a in the PD-1-negative population but this effect was less pronounced in pomalidomide-treated HIV-specific CD8^+^ T-cells (Fig. 2l). Within the degranulating populations, the expression of cytotoxic molecules was similar between the PD-1-expressing and PD-1 negative tetramer+ pool (Fig. 2m).

We then assessed the individual cytolytic capacity of the tetramer+ HIV-specific CD8^+^ T-cells using the HIV CD8^+^ Killing Assay, normalising for the input of tetramer^+^ HIV-specific CD8^+^ T-cells (effector) to HIV peptide-loaded CD4^+^ T-cells (target). Aligning with the intracellular cytokine profiling, we found pomalidomide treatment did not alter the cytolytic capacity of HIV-specific CD8^+^ T-cells on a per-cell basis (Fig. 2n), therefore showing that the increased killing capacity following pomalidomide treatment was a result of the expansion of HIV-specific cytotoxic CD8^+^ T-cells, rather than their individual killing capacity.

### Transcriptional regulators MYB and BATF3 are associated with the expansion of HIV-specific CD8^+^ T-cells following pomalidomide treatment

**Fig. 3:**
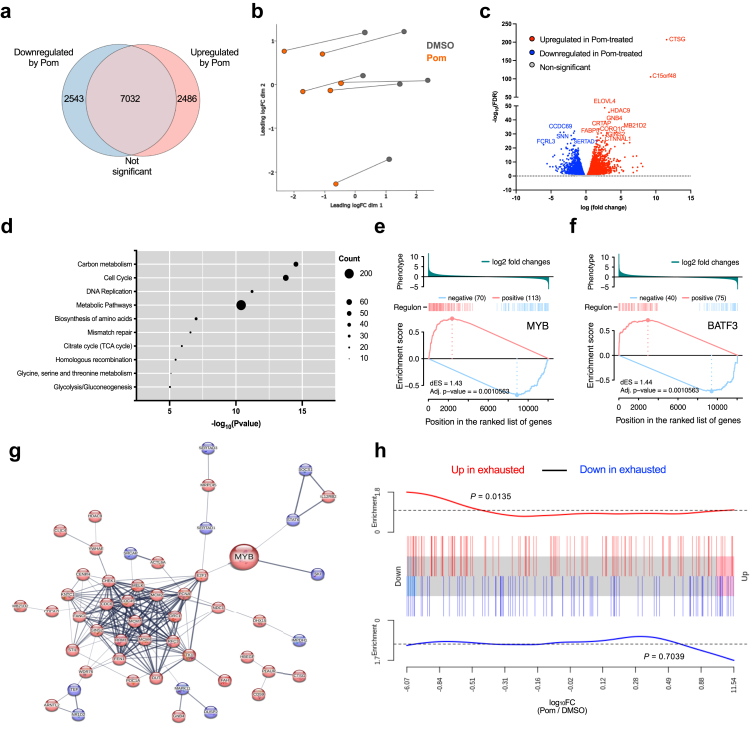
**Transcriptional regulators MYB and BATF3 are associated with pomalidomide-mediated expansion of less transcriptionally-exhausted HIV-specific CD8^+^ T-cells**. Tetramer^+^ HIV-specific CD8^+^ T-cells were sorted following 13-days expansion in the presence of DMSO or pomalidomide (0.25 μM) and sequenced using Illumina bulk RNA sequencing (n = 6). (**a**) Venn diagram shows the number of genes that were significantly upregulated, significantly downregulated, or not significantly changed in pomalidomide-treated tetramer^+^ HIV-specific CD8^+^ T-cells compared to DMSO-treated. Statistical significance determined as FDR < 0.05. (**b**) Multi-dimensional scaling (MDS) plot of tetramer-sorted, HIV-specific CD8^+^ T-cells. (**c**) Volcano plot showing differentially expressed genes (DEG) (false discovery rate (FDR) < 0.05) between pomalidomide-treated and DMSO-treated tetramer^+^ HIV-specific CD8^+^ T-cells, with the top 15 DEG annotated. (**d**) KEGG Pathway analysis on differentially expressed biomarkers (FDR <0.05), with the pathways that were upregulated at the highest significance levels in tetramer^+^ HIV-specific CD8^+^ T-cells following pomalidomide-treated conditions shown. Gene input count is shown by the size of the data point. (**e-f**) Two-tailed GSEA analysis showing the enrichment of MYB and BATF3 as potential transcriptional regulators of pomalidomide-mediated transcriptional effects in tetramer-sorted, HIV-specific CD8^+^ T-cells, based on Reconstruction of Transcriptional regulatory Networks and analysis of regulons (RTN) analysis. Vertical bars show the regulon's positive (red) or negative (blue) targets, ranked by differential gene expression (DEG) (phenotype). Lines show the running sum of regulon's positive (red) and negative (blue) targets, respectively, increasing in enrichment at the position of regulon's gene set among DEGs. dES calculated as the difference of the phenotype enrichment score (ES) of the positive and negative regulon, with a positive score reflecting activated regulon activity, and a negative value indicating a repressed regulon. The greater the positive score, the greater the induction and activation of the transcriptional regulator. (**g**) Protein–protein interaction (PPI) network in tetramer-sorted, HIV-specific CD8^+^ T-cells constructed by STRING database based on the top 200 DEG, and top 50 regulons implicated in pomalidomide's response. Proteins annotated, with the interconnecting line thickness indicating the strength of data support, based on known interactions from curated databases, or experimentally determined. Proteins shown are significantly upregulated (red), or significantly downregulated (blue). Of the DEG and regulons analysed, the 112 most relevant proteins shown. **(h)** Gene set enrichment analysis based on rotation gene set tests (ROAST). Barcode plots shows the enrichment of the gene set in a ranked gene list. Genes typically upregulated (red) and typically downregulated (blue) in the ‘core exhaustion profile’, plotted as the log_10_FC of pomalidomide-treated HIV-specific CD8^+^ T-cells relative to DMSO.

To investigate the transcriptional drivers behind the pomalidomide-mediated proliferation of HIV-specific CD8^+^ T-cells, HIV-specific tetramer^+^ CD8^+^ T-cells were sorted from six donors after 13 days of pomalidomide treatment in the presence of an HIV immunodominant peptide, and the transcriptional profile was evaluated using bulk RNA sequencing. Transcriptional analysis identified a large number of differentially expressed genes in the pomalidomide-treated cells compared to DMSO-treated controls (Fig. 3a), with multi-dimensional scaling (MDS) analysis revealing a highly distinct clustering of pomalidomide-treated tetramer^+^ HIV-specific CD8^+^ T-cells compared to DMSO controls (Fig. 3b). A consistent expression profile of the top 30 most significant genes was observed across the 6 donors (Figure S3a), with Cathepsin G (CTSG) and Chromosome 15 Open Reading Frame 48 (C15orf48) displaying the most dramatic upregulation (Fig. 3c).

Kyoto Encyclopedia of Genes and Genomes (KEGG) Pathway analysis revealed that whilst a large portion of differentially expressed genes were associated with metabolic pathways, pathways associated with carbon metabolism, cell cycle, and DNA replication were most strongly upregulated with pomalidomide treatment, with a clear upregulation of genes in the G1, S, G2 and M phase of the cell cycle, aligning with the greater proliferative profile observed in functional assays (Fig. 3d, S3b-e).

Using Reconstruction of Transcriptional regulatory Networks (RTN) analysis, various regulons were shown to be differentially expressed in pomalidomide-versus DMSO-treated HIV-specific CD8^+^ T-cells (Table 1). MYB and BATF3 were identified as two significantly enriched regulators in pomalidomide-treated HIV-specific CD8^+^ T-cells (Fig. 3e and f). In particular, MYB, a transcription factor that has been implicated in CD8^+^ T-cell stemness, polyfunctionality and survival, i.e. supporting the longevity of antigen-specific CD8^+^ T-cells,^42^ was one of the genes upregulated at the highest level by pomalidomide (3.56-fold; FDR = 3.84E-20), and may support the pro-proliferative profile observed, with protein–protein interaction (PPI) analysis highlighting the elevated expression of E2F1, a cell cycle effector shown to complex with MYB^43^ (Fig. 3g). Similarly, basic region-leucine zipper transcriptional factor ATF-like (BATF)-3 was upregulated (3.36-fold; FDR = 4.06E-18). BATF3 is an activator protein (AP)-1 that has been shown to support the survival, cellular fitness and memory formation of antigen-specific CD8^+^ T-cells by negatively regulating the proapoptotic protein, BIM.^44^ BATF3 expression has been previously demonstrated to regulate the expression of IL-2Rα/β in anaplastic large cell lymphoma,^45^ and indeed we found that IL2Ra was significantly upregulated in pomalidomide-treated HIV-specific CD8^+^ T-cells (3.25-fold, FDR = 3.94E-11). This was accompanied by a highly significant but small reduction in IL-2Rb (−0.8-fold; FDR = 2.90E-16) and a non-significant change in IL-2Rg (FDR = 0.322). These results provide insights into the potential drivers of HIV-specific CD8^+^ T-cells expansion following pomalidomide treatment, with changes in MYB and BATF3 regulons as well as elevated carbon metabolism, cell-cycle and DNA replication pathways.

**Table 1:** Top 20 regulons differentially expressed in pomalidomide-treated HIV-specific CD8^+^ T-cells.

Regulon	Regulon size	Adjusted P-value	dES	Name
TIGD6	153	0.0010563	1.57	Tigger Transposable Element Derived 6
ZNF85	129	0.0010563	1.46	Zinc Finger Protein 85
BATF3	115	0.0010563	1.44	Basic Leucine Zipper ATF-Like Transcription Factor 3
MYB	183	0.0010563	1.43	MYB Proto-Oncogene, Transcription Factor
ZNF148	140	0.0010563	−1.42	Zinc Finger Protein 148
SRCAP	110	0.0010563	−1.43	Snf2 Related CREBBP Activator Protein
ZBTB49	116	0.0010563	−1.44	Zinc Finger And BTB Domain Containing 49
STAT6	110	0.0010563	−1.44	Signal Transducer And Activator Of Transcription 6
KLF2	126	0.0010563	−1.45	KLF Transcription Factor 2
ZNF493	75	0.0010563	−1.45	Zinc Finger Protein 493
ZNF428	87	0.0010563	−1.45	Zinc Finger Protein 428
ZNF281	131	0.0010563	−1.46	Zinc Finger Protein 281
ZNF549	131	0.0010563	−1.46	Zinc Finger Protein 549
ZNF561	113	0.0010563	−1.46	Zinc Finger Protein 561
ZFP28	100	0.0010563	−1.47	ZFP28 Zinc Finger Protein
SKI	93	0.0010563	−1.49	SKI Proto-Oncogene
PLAGL1	93	0.0010563	−1.49	PLAG1 Like Zinc Finger 1
ZNF320	78	0.0010563	−1.5	Zinc Finger Protein 320
NR1D2	102	0.0010563	−1.51	Nuclear Receptor Subfamily 1 Group D Member 2
ZNF564	110	0.0010563	−1.58	Zinc Finger Protein 564

Regulons were evaluated in tetramer^+^ HIV-specific CD8^+^ T-cells sorted and sequenced from pomalidomide or DMSO-treated PBMC from ART-suppressed PLHIV. Using the Reconstruction of Transcriptional regulatory Networks (RTN) analysis, the most significant regulons were identified by intersecting the regulons with differentially expressed genes and running transcriptional network analysis (TNA).

To determine if the PD-1 downregulation in HIV-specific CD8^+^ T-cells following pomalidomide was reflective of an overall reversal of exhaustion, we compared the transcriptional profile of pomalidomide-expanded tetramer-positive CD8^+^ T-cells to a transcriptional ‘core exhaustion profile’ of CD8^+^ T-cells, which included genes implicated in cellular differentiation, effector function, and senescence.^46^ The transcriptional profile of pomalidomide-treated HIV-specific CD8^+^ T-cells showed a downregulation of genes classically upregulated in exhaustion (p = 0.0135; Fig. 3h, Figure S3f and g), thus demonstrating that the reduced expression of ICs, in particular PD-1, in pomalidomide-expanded HIV-specific CD8^+^ T-cells occurred concurrently with a reduction in features of exhaustion at the transcriptional level.

Taken together, pomalidomide was found to reduce the transcriptional exhaustion profile of HIV-specific CD8^+^ T-cells, notably reducing PD-1 expression, enhancing their proliferative capacity following exposure to HIV antigen, culminating in greater killing of HIV-expressing CD4^+^ T-cells. These functional changes were largely related to improved proliferative capacity and did not extend to any change in the cytolytic capacity of each cell.

### IL-2 contribution to pomalidomide's pro-proliferative effects

Given that pomalidomide induces T-cell production of IL-2,^18^ we also compared the functional properties of pomalidomide to high-dose exogenous IL-2 (100 IU/mL). Whilst high-dose exogenous IL-2 was associated with marginally improved viability (Figure S4a), pomalidomide induced greater expansion of HIV-specific tetramer+ CD8^+^ T-cells than exogenous IL-2 (Figure S4b and c), and this led to greater killing of HIV-peptide-loaded CD4^+^ T-cells in the HIV-specific CD8^+^ T-cell killing assay when normalised to total CD8^+^ T-cells (Figure S4d and e). Neither treatment with pomalidomide nor exogenous IL-2 induced a change in the individual cytotoxic capacity of HIV-specific CD8+ T-cells (Figure S4f). Taken together, whilst the expansion of HIV-specific CD8^+^ T-cells and increased HIV-specific lysis can in part be explained by IL-2 mediated effects, pomalidomide confers additional beneficial effects on T-cell function compared to IL-2 alone.

### Pomalidomide enhances NK cell-mediated killing of productive HIV-infected cells in a ligand-dependent manner

**Fig. 4:**
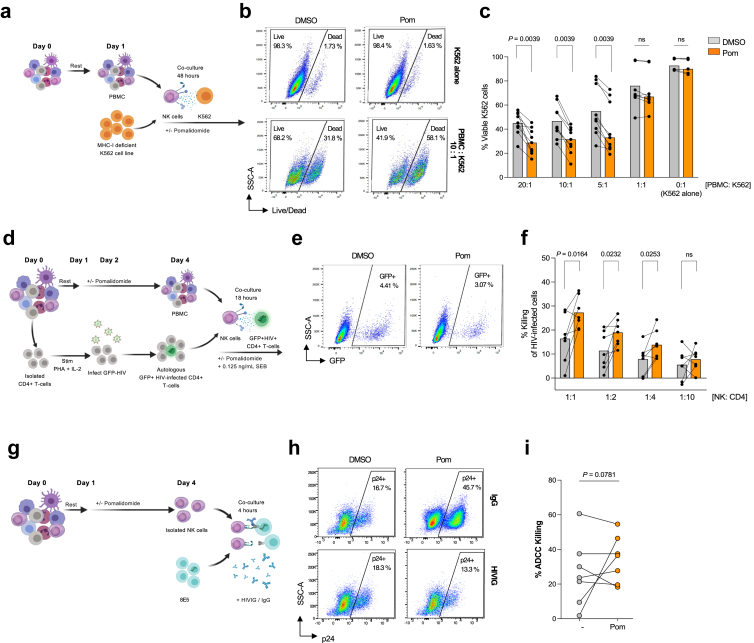
**Pomalidomide enhances direct NK cell killing of productive HIV-infected CD4^+^ T-cell**s. PBMC from PLHIV or HIV-negative individuals were treated with pomalidomide at 0.25 μM, or DMSO, *ex vivo*. (**a**) Schematic of K562 assay used to measure direct NK cell cytotoxicity of treated NK cells. PBMC from PLHIV were co-cultured with target labelled-K562 cells for 48 h in the presence of DMSO or pomalidomide, at various effector:target ratios (PBMC: K562); (**b**) Representative gating strategy with (**c**) K562 cell viability measured at 48 h as a measure of NK cell cytotoxicity (n = 9). (**d**) NK cell killing of HIV-infected cells was measured using purified DMSO- or pomalidomide-treated NK cells from HIV-negative donors co-cultured with autologous *in vitro* infected CD4^+^ T-cells (infected with an GFP-reporter R5-tropic HIV) for 18 h with continued exposure to drug conditions. Culturing was in the presence of low-level antigen stimulation with low-dose staphylococcal enterotoxin B (SEB). (**e**) Flow plots representative of gating used to measure the relative loss of GFP-expressing CD4^+^ T-cells. (**f**) Bar graph shows the percentage of NK cell-mediated killing of HIV-infected CD4^+^ T-cells (n = 7). (**g**) PBMC from PLHIV were treated with pomalidomide at 0.25 μM, or DMSO *ex vivo,* and evaluated in the ADCC Assay, as shown. PBMC from PLHIV were treated with DMSO or pomalidomide for 72 h, and purified NK cells were co-cultured with eFluor670-labelled LAV/8E5 target cells, in the presence of anti-HIV immunoglobulin (HIVIG) or IgG isotype control. (**h**) Representative flow plot of the p24 gating strategy (gated on Live^+^ efluor670-labelled LAV/8E5 target cells), with ADCC measured as the relative loss of p24-positive 8E5 target cells. (**i**) Percentage of anti-HIV ADCC following DMSO or pomalidomide treatment for 72 h (n = 7) (Fig. 4c–i significance determined by Wilcoxon matched-pairs signed rank test, bars showing median + IQR. Fig. 4f significance determined by Paired T-test, bars showing mean +SEM. ∗p < 0.05, ∗∗p < 0.01; ns, not significant).

In addition to the effects on HIV-specific CD8^+^ T-cells, we next investigated whether pomalidomide impacts NK cell cytotoxic function against MHC-I deficient cells and HIV-infected CD4^+^ T-cells. To directly assess NK cell cytotoxicity, we co-cultured PBMC isolated from PLHIV on ART with fluorescently-labelled K562 target cells at various effector:target (E:T) ratios, in the presence of pomalidomide (0.25 μM) or DMSO (Fig. 4a and b). K562 is a cell line that lacks MHC-I expression and therefore acts as a target for NK cell killing but not for CD8^+^ cytotoxic T-cells. Following 48 h of pomalidomide treatment, NK-mediated killing activity increased compared to DMSO, with a relative increase in the lysis of K562 cells by 34% (p = 0.0039), 34% (p = 0.0039), and 26% (p = 0.0039) at 20:1, 10:1, and 5:1 E:T ratios (PBMC: K562), respectively (Fig. 4c).

We then investigated the capacity of pomalidomide-treated NK cells to selectively eliminate HIV-infected autologous CD4^+^ T-cells through receptor–ligand interactions. Using PBMCs from HIV-negative individuals, CD4^+^ T-cells were infected with a CCR5-tropic laboratory HIV isolate (AD8) that expressed green fluorescent protein (GFP) and co-cultured with autologous NK cells pre-treated with pomalidomide or DMSO, in the presence of low-dose antigen co-stimulation (Fig. 4d and e). Low-dose staphylococcal enterotoxin B (SEB) was used to provide a secondary activation of the T-cell receptor, as described in previous studies of HIV-negative donors.^39^^,^^47^^,^^48^ Pomalidomide at 0.25 μM significantly enhanced killing of HIV-infected target cells, measured as a reduction in GFP expression, with a median increase in killing capacity of 1.87-fold (p = 0.0164) at 1:1 and 1.36-fold (p = 0.0232) at 1:2 E:T ratios of NK cells: CD4^+^ T-cells (Fig. 4f).

To investigate the capacity of pomalidomide-treated NK cells to deplete HIV-infected cells through antibody-dependent cellular cytotoxicity (ADCC), we employed the infected cell elimination assay as previously described.^37^ In brief, pomalidomide pre-treated NK cells were cultured with the untreated HIV-infected cell line, LAV/8E5, in the presence of immunoglobulin from PLHIV (HIVIG) or IgG isotype control (Fig. 4g and h). We found that pomalidomide did not significantly increase anti-HIV ADCC (p = 0.0781; Fig. 4i). Taken together, this indicates that pomalidomide supports NK cell clearance of HIV-infected cells in a ligand-dependent manner but does not enhance ADCC-mediated clearance.

### Pomalidomide expands cytotoxic CD56^dim^ CD16^+^ NK cells and induces TIGIT downregulation to support NK cell cytotoxicity

**Fig. 5:**
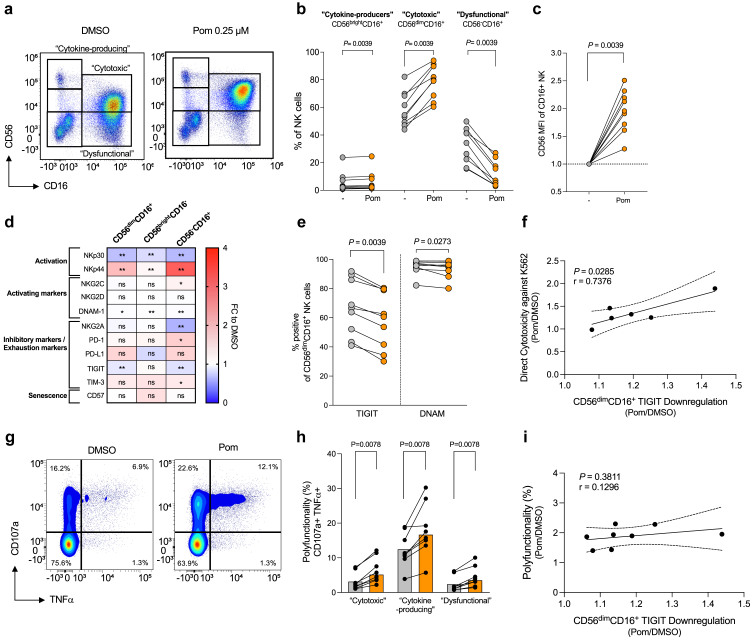
**Pomalidomide reduces NK cell dysfunction and improves NK cell polyfunctionality**. PBMC from PLHIV were treated with pomalidomide at 0.25 μM, or DMSO, *ex vivo* (n = 9). (**a**) Representative flow plot of the gating strategy for NK cell subsets (pre-gated on live CD3-CD14-CD19-CD40-CD80-) in DMSO and pomalidomide-treated PBMC following 72 h culture. (**b**) Percentages of NK cell subsets to the overall NK cell pool. (**c**) The median fluorescence index (MFI) of CD56 in CD16-expressing NK cells of pomalidomide-treated relative to DMSO. (**d**) Heatmap with the median fold change (FC) in surface marker expression on NK cell subsets. (**e**) Frequency of CD56^dim^CD16^+^ “cytotoxic” NK cell subset expressing TIGIT or DNAM. (**f**) Correlation between enhanced direct NK cytotoxicity in K562 assay following pomalidomide treatment (FC relative to DMSO at 20:1 effector:target ratio) and TIGIT downregulation in CD56^dim^CD16^+^ NK cell subset (FC pomalidomide relative to DMSO) (n = 6). (**g**) PBMC from PLHIV were treated *ex vivo* with DMSO or pomalidomide for 72 h and cultured for a further 4 h with K562 target cells in the presence of monensin and brefeldin. Representative flow plots of CD107a and TNFα expression. (**h**) Frequency of polyfunctional (CD107a^+^TNFα^+^) cells in NK cell subsets (n = 8). (**i**) Correlation between polyfunctionality following pomalidomide treatment (FC relative to DMSO) and TIGIT downregulation in CD56^dim^CD16^+^ NK cell subset following pomalidomide treatment (FC relative to DMSO) (n = 8). (Wilcoxon matched-pairs signed rank test, with bars showing median + IQR. Fig. 5f–i correlation and significance determined using Pearson correlation, with 95% confidence intervals shown).

To elucidate the mechanisms underpinning the improved NK cell clearance in a direct ligand-dependent manner, we phenotyped NK cells in samples from PLHIV treated with pomalidomide *ex vivo* for 72 h. Pomalidomide did not alter the proportions of CD4^+^ T-cells, CD8^+^ T-cells, NK cells, monocytes, dendritic cells, or B cells within the PBMC population (Figure S5a), but strongly upregulated CD56 expression leading to a change in the distribution of NK cell subsets (p = 0.0039; Fig. 5a–c). NK cells are classically characterised by their expression of CD56 and CD16 with CD56^bright^CD16^-^ considered immature, “cytokine-producing” NK cells, whereas CD56^dim^CD16^+^ are considered the matured “cytotoxic” NK cells.^49^ The CD56^−^CD16^+^ NK cell subset is considered “dysfunctional” and is expanded in ART-suppressed PLHIV.^9^^,^^10^^,^^50^ Pomalidomide significantly reduced the CD56^−^CD16^+^ NK cell subset population by 3.0-fold (p = 0.0039), with a corresponding significant increase in the proportion of the CD56^dim^CD16^+^ cytotoxic NK cell subset (p = 0.0039; Fig. 5b).

Within all NK cell populations, the NK cell activation marker, NKp44, was upregulated (p = 0.0039), with a concomitant reduction in the activation marker, NKp30 (p = 0.0039; Fig. 5d). Similar to CD8^+^ T-cells, pomalidomide reduced the expression of TIGIT, an inhibitory receptor for CD155/PVR that exerts negative regulation of CD8^+^ T and NK cell functional activity, on CD56^dim^CD16^+^ and CD56^−^CD16^+^ NK cells (p = 0.0039 and p = 0.0078, respectively; Fig. 5d and e). Given that TIGIT-mediated signalling in the TIGIT/DNAM-1/CD155 axis is moderated by DNAM-1 expression, we also investigated DNAM-I expression. We found a minor but statistically significant reduction in DNAM-1 on CD56^dim^CD16^+^ “cytotoxic” NK cells in the presence of pomalidomide compared to DMSO (p = 0.0273; Fig. 5d and e), but this was predominately within the TIGIT-expressing CD56^dim^CD16^+^ NK cells population with no significant change in DNAM-1 expression in the TIGIT-negative CD56^dim^CD16^+^ NK cell population (Figure S5b). Furthermore, a positive correlation between enhanced NK cell-mediated killing of K562 cells and TIGIT downregulation in the cytotoxic CD56^dim^CD16^+^ NK cell subset was observed (p = 0.0285; Fig. 5f), indicating that pomalidomide-driven TIGIT downregulation in the CD56^dim^CD16^+^ NK cell subset may support the pro-cytotoxic DNAM-1-CD155 interaction and enhance NK cell cytotoxicity.

Taken together, these results indicate that pomalidomide induces phenotypic changes that favour the expansion of NK cells with greater cytotoxic potential, characterised by lower expression of the inhibitory receptor, TIGIT.

### Pomalidomide confers enhanced NK cell polyfunctionality

To further clarify the mechanisms underpinning the improved NK cell killing profile following pomalidomide treatment, we quantified the induction of degranulation, and effector cytokines in the CD56^dim^CD16^+^ cytotoxic NK cell subset to determine if the altered phenotype conferred enhanced NK cell cytotoxicity. Polyfunctional NK cells were defined by co-expression of CD107a and TNFα^51^ (Fig. 5g). Pomalidomide treatment of PBMC significantly enhanced CD56^dim^CD16^+^ cytotoxic NK cell polyfunctionality by 1.9-fold when exposed to K562 cells (p = 0.0078; Fig. 5h). Both CD56^−^CD16^+^ and CD56^bright^CD16^-^ NK cell populations also demonstrated an increase in polyfunctionality with pomalidomide treatment (p = 0.0078 and p = 0.0078, respectively). There was, however, no correlation observed between the observed TIGIT downregulation and the improved polyfunctionality within the CD56^dim^CD16^+^ cytotoxic NK cells following pomalidomide treatment (p = 0.3811; Fig. 5i), suggesting these two mechanisms operate independently to support NK cell cytotoxicity.

Collectively, these data demonstrate that pomalidomide enhanced NK cell polyfunctionality in cells from PLHIV on ART treated *ex vivo*, enhanced NK cell cytotoxicity by reducing NK cell dysfunction and exhaustion and expanded an NK cell subset with enhanced cytotoxic potential that can readily lyse HIV-infected cells. The combined effects of pomalidomide on NK cell cytotoxicity and HIV-specific CD8^+^ T-cell immunity provide pre-clinical support for pomalidomide to be tested as an immunotherapeutic intervention to induce immune control of HIV replication in the absence of ART.

## Discussion

Interventions capable of reinvigorating innate and adaptive anti-HIV immunity are of great interest in achieving an HIV cure.^11^ Using PBMCs from PLHIV on ART, we demonstrated that pomalidomide can expand HIV-specific CD8^+^ T-cells and increase the killing of peptide-loaded target cells. Pomalidomide treatment resulted in the activation of CD8^+^ T-cells, expanded non-exhausted HIV-specific CD8^+^ T-cells and enhanced killing of peptide-loaded target cells. In transcriptional analyses, we identified MYB and BATF3 as potential regulators of the expansion of HIV-specific CD8^+^ T-cells. Importantly, we saw no impact of pomalidomide on the proliferation of CD4^+^ T-cells, which could be a concern for an HIV cure intervention. Pomalidomide also increased cytotoxic NK cells with enhanced polyfunctionality, leading to augmented killing of HIV-infected target cells. Together, these findings provide pre-clinical support for pomalidomide to be tested as an immunotherapeutic intervention to induce immune control of HIV replication in the absence of ART.

This study presents a comprehensive examination of the immune-enhancing antiviral effects of pomalidomide. Pomalidomide has clinical efficacy against multiple myeloma, Kaposi Sarcoma, and high-grade squamous intraepithelial lesions.^38^^,^^39^^,^^52^, ^53^, ^54^ In clinical trials of PLHIV receiving pomalidomide for the treatment of certain malignancies, the HIV-specific immune effects have not previously been investigated.^19^^,^^52^^,^^53^^,^^55^ Only one prior study evaluated the HIV-specific immune-enhancing effects of pomalidomide *in vitro*, demonstrating an increase in polyfunctional T-cell responses following HIV-peptide stimulation in sorted CD8^+^ T-cells from PLHIV.^20^ In our study, we sorted HIV-specific tetramer^+^ cells from PLHIV on ART and were therefore able to characterise the impact of pomalidomide on HIV-specific T-cell proliferation and cytotoxic capacity. We found that pomalidomide improved HIV-specific killing and upregulated MYB and BATF3, which have been identified as potential transcriptional regulators that could enhance the proliferative capacity of HIV-specific CD8^+^ T-cells. It is important to highlight that we performed *ex vivo* examination of pomalidomide using PBMC, not purified CD8^+^ T-cells, as we hypothesised that pomalidomide may work through autocrine and paracrine mechanisms, stimulating NK cells and T-cells as well as altering monocyte metabolism.^56^, ^57^, ^58^, ^59^

We showed that pomalidomide both activated CD8^+^ T-cells and induced a greater expansion of HIV-specific CD8^+^ T-cells, compared to tetramer- CD8^+^ T-cells, but was not accompanied by an expansion of CD4^+^ T-cells. Whether the effects on tetramer^+^ cells were HIV-specific cannot be definitively concluded from our experiments, but we speculate that pomalidomide does not operate in an HIV-specific manner. Given the greater proliferation of HIV-specific tetramer+ compared to tetramer- CD8^+^ T-cells following stimulation with pomalidomide and HIV antigen, and its synergistic activation with antigen exposure, it is possible that pomalidomide may also confer similar pro-proliferative effects on other antigen-specific CD8^+^ T-cells in the presence of cognate antigens. These effects are likely, in part, due to the increase in IL-2 that accompanies pomalidomide treatment and IL-2R signalling implicated in elevating the expression of the pro-survival proteins, B-cell lymphoma-2 (Bcl-2) and X-linked inhibitor of apoptosis proteins (XIAP).^60^^,^^61^ These changes will subsequently skew the activation responses of antigen-specific CD8^+^ T-cells following antigen exposure towards proliferation rather than activation-induced cell death.^62^^,^^63^ Indeed, we recently demonstrated that pomalidomide treatment paradoxically elevated Bcl-2 expression in CD4^+^ T-cells, supporting cell survival.^22^ Hence, in the context of antigen-specific CD8^+^ T-cell immunity, we propose that pomalidomide-mediated increase in IL-2 may offset the pro-apoptotic features of pomalidomide, contributing to enhanced immune responses with potentially greater longevity.

The effects of pomalidomide on HIV-specific CD8^+^ T-cells were accompanied by a reduction in surface PD-1 expression. Increased PD-1 expression on HIV-specific CD8^+^ T-cells has been identified as a key driver of HIV-associated T-cell exhaustion^3^^,^^64^ and blocking of the PD-1-PD-L1 pathway in murine models restored virus-specific CD8^+^ T-cell proliferative and cytolytic capacity.^64^^,^^65^ Anti-PD-1 agents have also been shown to reinvigorate HIV-specific cells *in vivo* in animal models,^66^ and in PLHIV.^67^^,^^68^ Whether the pomalidomide-induced reduction in PD-1 expression could provide a similar favourable functional effect *in vivo* is not clear, but we note that the primary effect on CD8-mediated lysis of HIV-expressing cells in our study was driven by an expansion of the total number of HIV-specific CD8^+^ T-cells, rather than an increase in per-cell cytotoxicity.

We showed that pomalidomide also induced expression of MYB and BATF3. MYB is associated with CD8^+^ T-cell stemness and polyfunctionality, and has been shown to increase the survival of memory CD8^+^ T-cells in mice.^42^^,^^69^ BATF3 has also been shown to contribute to the recall and longevity of antigen-specific CD8^+^ T-cells,^44^ and may regulate the expression of IL-2αβ.^45^ Elevated transcription of IL-2Rα on sorted HIV-specific CD8^+^ T-cells with pomalidomide treatment likely contributes to the effects observed, with IL-2Rα shown to sustain IL-2 signalling independently of IL-2Rβγ by promoting IL-2 recycling back to the cell surface whilst also generating an extracellular reservoir to preserve IL-2 in an IL-2 deficient environment.^70^ A recent study that combined a low-affinity IL-2 coupled to an anti-PD-1 antibody successfully enhanced anti-tumour immunity by tumour-infiltrating lymphocytes,^71^ and the immunological mechanisms that likely underpin pomalidomide-associated immune enhancement are not dissimilar, further implicating the BATF3/IL2-Rα/IL-2/PD-1 pathway in the effects observed on HIV-specific CD8^+^ T-cells in the presence of cognate HIV antigen. Taken together, the upregulation of MYB and BATF3 by pomalidomide may be a key factor leading to the enhanced HIV-specific CD8^+^ T-cell proliferation and killing that we observed in this study.

We acknowledge that our findings implicating MYB and BATF3 as key regulators in the pomalidomide-induced effects on HIV-specific CD8^+^ T-cells were based on, and limited to, RTN analysis. Ideally, these observations would be validated in knockout models; however, MYB loss in a *c-myb* knockout murine model impeded the formation of stem cell-like T_CM_ CD8^+^ T-cells with loss of Bcl-2 and massive apoptosis of CD8^+^ T-cells in the initial immune response to infection.^42^ This aligns with another study, where the establishment of lymphocytic choriomeningitis virus (LCMV)-chronic infection in mice lacking MYB in T cells (*Myb*^*fl/fl*^*Cd4*^*Cre*^) led to severe immunopathology, with mice typically becoming moribund within 10 days post-infection.^69^ Similarly, BATF3 knockout mice (*Batf3*^*−/−*^*OT-I Rag1*^*−/−*^*)* exhibited greater apoptosis of CD8^+^ T-cells during contraction, disrupting the memory development and recall of CD8^+^ T-cells.^72^ These phenotypes that accompany loss of MYB and BATF3 constrain the evaluation of memory CD8^+^ T-cell responses in murine knockout models and limit subsequent studies of immunotherapeutic agents, such as pomalidomide, on antigen-specific CD8^+^ T-cell responses. Additionally, while overexpression models have been valuable for elucidating the regulatory roles of MYB and BATF3 in CD8^+^ T-cell memory responses,^42^^,^^44^^,^^69^^,^^73^ these profiles may differ in HIV-specific CD8^+^ T-cell responses and warrant further investigation.

We also identified significant changes in NK cells following pomalidomide treatment *ex vivo*, which were dominated by an expansion of CD56^dim^CD16^+^ “cytotoxic” NK cells and an increase in production of effector cytokines, leading to greater killing of NK cell targets. These results are consistent with findings from clinical trials of lenalidomide-refractory myeloma, where it was shown that pomalidomide in combination with dexamethasone increased CD56, CD16, NKG2D, and TIM-3 expression on NK cells and enhanced NK cell production of TNFα and IFNγ.^18^ Importantly, we demonstrated that these changes had a significant functional impact with increased NK cell clearance of HIV-infected CD4^+^ T-cells.

The immune-enhancing effects of pomalidomide on NK cells have previously been thought to result from increased IL-2 production following the ubiquitination and degradation of the zinc finger transcription factors, Ikaros and Aiolos.^56^^,^^57^^,^^74^ It is likely that the changes we observed in NK cells from PLHIV following pomalidomide *ex vivo* were driven by local IL-2 production. IL-2 can activate NK cells isolated from PLHIV on ART and upregulate the NK cell activation marker, NKp44, without inducing NKp30,^75^ similar to what was observed in our study with pomalidomide. Consistent with these findings, increased NKp44 expression can be used as a proxy for NK cell responsiveness to IL-2.^76^

Although pomalidomide has been shown in previous studies to enhance ADCC,^77^^,^^78^ we were unable to demonstrate such an effect in our study. Potential explanations for our findings could be related to the assay design and our use of pooled HIV immunoglobulin, rather than a specific neutralising antibody, or as a result of there being no change in the expression of CD16, a key modulator of NK-mediated ADCC.^22^

While this study provides a comprehensive assessment of the impact of pomalidomide on HIV-specific CD8^+^ T-cell and NK cell function *in vitro/ex vivo*, we recognise several limitations. First, all donors living with HIV were Caucasian men, and HIV-specific CD8^+^ T-cell responses were only analysed in a restricted number of HLA-types that were compatible with the available MHC-I:HIV-peptide tetramers. Further investigation would be required to determine whether our findings extend to other common HLA types and in women and PLHIV with different ethnicities. Second, treatment with pomalidomide *ex vivo* and *in vitro* may not reflect *in vivo* effects, however, we mimicked more complex paracrine and autocrine effects by treating PBMC with pomalidomide and then isolating HIV-specific CD8^+^ T-cells and NK cells. Third, we evaluated pomalidomide at a concentration corresponding to the maximal plasma concentration achieved with 5 mg daily dosage.^23^ The efficacy of pomalidomide at lower concentrations may be relevant for clinical development but was not explored in this study. Fourth, we evaluated the effect of pomalidomide on HIV-specific CD8^+^ T-cells using HIV-specific tetramers, but did not assess other antigen-specific CD8^+^ T-cells. Finally, we assessed the effects on HIV-specific killing using productively infected cells or HIV-peptide loaded cells, which does not reflect the challenges of eliminating latently infected cells which express minimal HIV antigen in the setting of viral suppression by ART.^79^ In addition, latently-infected cells are more resistant to killing through upregulation of the pro-survival proteins, B-cell lymphoma (Bcl)-2 and Baculoviral IAP Repeat Containing (BIRC)-5, as well as increased expression of immune checkpoints including PD-1, TIGIT, and various other immune evasion markers,^80^, ^81^, ^82^ which may not be fully recapitulated in our target cells.

Taken together, the observed pomalidomide-induced enhancement of HIV-specific CD8^+^ T-cell expansion and NK cell cytotoxicity, in the absence of increased per-cell cytotoxicity of HIV-specific CD8^+^ T-cells, positions pomalidomide as a promising immune-enhancing therapeutic. The lack of change in HIV-specific CD8^+^ T-cell per-cell cytotoxicity should not discount the potential benefits of pomalidomide in supporting the recall of anti-HIV immunity. Furthermore, the augmented proliferative capacity of HIV-specific CD8^+^ T-cells led to greater clearance of HIV-target cells in our CD8^+^ T-cell killing assay. This finding is particularly relevant to the development of strategies aimed at inducing ART-free viral control, as superior viral suppression in HIV controllers has been linked to greater stem-like memory features and enhanced recall capacity of HIV-specific CD8^+^ T-cells.^83^ HIV-specific CD8^+^ T-cell proliferative capacity has been inversely correlated with viral load in ART-naïve PLHIV and elite controllers,^84^^,^^85^ greater SIV viral control post-ART,^86^ and in post-treatment controllers.^87^ Hence, pomalidomide and its pro-proliferative features on HIV-specific CD8^+^ T-cells may offer a promising avenue to reinvigorate HIV-specific immunity.

In conclusion, we demonstrated that pomalidomide enhanced NK cell and HIV-specific CD8^+^ T-cell immune responses through the expansion of HIV-specific CD8^+^ T-cells and increasing the proportion of functional and cytolytic NK cells to boost killing of HIV-infected target cells. Transcriptomic analyses identified MYB and BATF3 as potential key regulators which may underlie the pomalidomide-associated enhanced expansion of HIV-specific CD8^+^ T-cells. These findings provide pre-clinical data to support further investigation of pomalidomide as an immune-enhancing therapeutic agent, alone or in combination with other agents, for enhanced immune control of HIV in the absence of ART.

## Contributors

R.D.P., T.A.R. and S.R.L. conceptualised the study; R.D.P., T.A.R., C.G., J.J.C., D.T.U., A.B., W.S.L., J.S., C.Y.C. and J.A. contributed to the study design and methodology; R.D.P., C.G., J.S. and A.S. performed the experiments and analysed and interpreted the data; R.D.P wrote the original manuscript draft; R.D.P., T.A.R., S.R.L., D.T.U., A.B. and W.S.L. reviewed and edited the manuscript; T.A.R., S.R.L. and R.D.P. received funding for the project; S.R.L., J.H.M., W.S.L., A.B. and A.S. contributed resources to the project. R.D.P., C.G. and T.A.R. have accessed and verified the underlying data. All authors reviewed and approved the final version of the manuscript.

## Data sharing statement

The sequencing data are available at the NCBI GEO (http://www.ncbi.nlm.gov/geo) under the accession number GSE244148. This paper does not report original code. All data reported in this paper will be shared with publication by the lead contact upon reasonable request and following the establishment of a material and data transfer agreement between institutions.

## Declaration of interests

R.D.P has received funding from the Melbourne HIV Cure Consortium. J.S and J.A has received funding from the J & M Wright Foundation, Australia and the Computational Sciences Initiative Committee. T.A.R has received funding from the Independent Research Fund, Denmark, Novo Nordisk Foundation, Viiv Healthcare, Gilead Sciences, and Abbvie outside the submitted work. S.R.L has received grant funding from the National Health and Medical Research Council of Australia, Australian Centre for HIV and Hepatitis Virology Research (ACH4), and funding for participation in scientific advisory boards from Gilead Sciences, ViiV, Abbvie, Immunocore, Vaxxinity, Biotron, First Health, and Esfam outside the submitted work. The authors have no additional conflicting financial interests.

## Appendix A Supplementary data

Supplementary Figure S1 **Pomalidomide supports the proliferation of tetramer-negative CD8^+^ T-cells but to a lesser extent than tetramer^+^ HIV-specific CD8^+^ T-cells in the presence of HIV antigen**. PBMC from PLHIV were pre-stained with the proliferation dye, CTV, and treated with DMSO or pomalidomide (0.25 μM) for 13 days, following exposure to an immunodominant HIV peptide. HIV-specific CD8^+^ T-cells were measured using a tetramer to the same immunodominant HIV peptide. (**a**) Validation of MHC-I:HIV tetramers, measuring degranulation (CD107a) and IFNγ in HIV-specific tetramer^+^ (pink) and tetramer- (grey) CD8^+^ T-cells (n = 4). (**b**) Frequency of viable PBMC after 13 days culture with pomalidomide (0.25 μM) or DMSO (n = 7). (**c**) Correlation between the baseline tetramer^+^ HIV-specific CD8^+^ T-cell frequency of total CD8^+^ T-cells and the pomalidomide-associated expansion of tetramer^+^ HIV-specific CD8^+^ T-cells (shown as fold change relative to DMSO). **(d**) Contribution of tetramer^+^ and tetramer- CD8^+^ T-cells to the live PBMC pool, shown as the fold change of pomalidomide-treatment relative to DMSO-treated conditions (**e)** Pie charts showing the median expression of CD8^+^ T-cell subsets within tetramer^+^ and tetramer- CD8^+^ T-cell populations. (**f**) Representative histogram of CTV division within tetramer^+^ HIV-specific CD8^+^ T-cells during 13-day culture with DMSO (grey) and pomalidomide (orange). (**g**) The frequency of tetramer-positive HIV-specific CD8^+^ T-cells (full circles, left y-axis), and the expression of PD-1 within the proliferating HIV-specific CD8^+^ T-cell population (circle outline, right y-axis) was quantified on days 4, 7, 10, and 13. DMSO indicated in grey, and pomalidomide-treated indicated in orange. Each plot represents a single donor tested. (**h**) Representative flow plot of the gating strategy for proliferation in DMSO and pomalidomide-treated HIV-specific CD8^+^ T-cells. (**i**) The MFI of PD-1 expression within the non-proliferating population, the lowly-, and highly-proliferative population, measured on day 13 in the DMSO and pomalidomide-treated conditions. (**j**) Frequency of cells that underwent proliferation following treatment with pomalidomide or DMSO, as determined by loss of CTV fluorescence index at the timepoint measured. Proliferation was measured in tetramer^+^ HIV-specific CD8^+^ T-cells, tetramer^-^ CD8^+^ T-cells, and CD8^-^ T-cells, which are predominately CD4^+^ T-cells. (**k**) Frequency of immune checkpoints (IC) markers/exhaustion populations of tetramer- TEM CD8+ T-cells in DMSO or pomalidomide-treated conditions. (n = 7; significance was determined using the Wilcoxon matched-pairs signed-rank test. ∗p < 0.05, ∗∗p < 0.01; ns, not significant).

Supplementary Figure S2 **Pomalidomide-associated enhanced proliferative capacity had no impact on the lytic capacity of HIV-specific CD8^+^ T-cells**. PBMC from PLHIV were treated with DMSO or pomalidomide (0.25 μM) for 13 days, following exposure to an immunodominant HIV peptide. PBMC were then re-stimulated with the same HIV peptide to measure cytotoxic molecule production. (**a**) Representative histograms and median fluorescence index (MFI) expression of cytotoxic molecules and cytokines in degranulating (CD107a+) HIV-specific CD8^+^ T-cells. (**b-c**) Upset plots representing the major co-expression profiles of cytotoxic molecules within the degranulating fraction of (b) DMSO-treated and (c) pomalidomide-treated tetramer^+^ HIV-specific CD8^+^ T-cells. Intersection size reflects the frequency of the population co-expressing the indicated cytotoxic molecules listed below. Set size represents the expression frequency of the singular cytotoxic molecule. (n = 7; significance determined using Wilcoxon matched-pairs signed rank test; ns, not significant).

Supplementary Figure S3 **Pomalidomide reduces the core exhaustion profile of HIV-specific CD8^+^ T-cells whilst promoting cell cycle progression**. Tetramer^+^ HIV-specific CD8^+^ T-cells were sorted and sequenced using Illumina bulk RNA sequencing (n = 7). (**a**) Heatmap showing the top 30 significant differentially expressed genes (DEG) in pomalidomide-treated (0.25 μM) HIV-specific CD8^+^ T-cells relative to DMSO-treated HIV-specific CD8^+^ T-cells. Statistical significance determined as FDR<0.05. (**b**) Heatmap of the genes implicated in carbon metabolism in KEGG pathway analysis, with DEGs from pomalidomide-treated HIV-specific CD8^+^ T-cells, relative to DMSO, shown. (**c**) Protein interaction networks implicated in carbon metabolism. Analysis based on KEGG pathway results, performed using protein interactions generated and visualised using Cytoscape. Genes significantly upregulated by pomalidomide highlighted in red. (**d**) Heatmap of the genes implicated in cell cycle, according to KEGG pathway analysis, with DEGs from pomalidomide-treated HIV-specific CD8^+^ T-cells, relative to DMSO, shown. (**e**) Protein interaction networks implicated in cell cycle. Analysis based on KEGG pathway results, performed using protein interactions generated and visualised using Cytoscape. Genes significantly upregulated by pomalidomide highlighted in red. (**f-g**) Heatmaps of the significantly altered ‘core exhaustion’ genes in pomalidomide-treated HIV-specific CD8^+^ T-cells relative to DMSO. (F) Genes typically upregulated in exhaustion, based on the ‘core exhaustion profile’. (G) Genes typically downregulated in exhaustion, based on the ‘core exhaustion profile’.

Supplementary Figure S4 **Comparison of pomalidomide to high-dose IL-2 on the expansion and killing capacity of HIV-specific CD8^+^ T-cells**. PBMC from PLHIV were treated with DMSO, pomalidomide (0.25 μM) or high-dose IL-2 (100 IU/mL) for 13 days, following exposure to an immunodominant HIV peptide. (**a**) Frequency of viable PBMC after 13 days culture with DMSO, pomalidomide or high-dose IL-2. (**b**) Frequency of tetramer^+^ HIV-specific CD8^+^ T-cells of CD8^+^ T-cell pool. (**c**) Absolute number of tetramer^+^ HIV-specific CD8^+^ T-cells. (**d**) Killing capacity was measured using the CD8^+^ T-cell killing assay. PBMC from PLHIV were stimulated with a HIV-immunodominant peptide and treated with DMSO or pomalidomide for 13 days. Purified CD8^+^ T-cells were co-cultured with autologous CD4^+^ T-cells stained with two CTV concentrations, with the lower concentration loaded with the HIV immunodominant peptide. HIV-specific lysis was calculated as the relative killing of the peptide-loaded CD4^+^ T-cells to the non-peptide-loaded CD4^+^ T-cells. Representative flow gating of the peptide-loaded CD4^+^ T-cells (HIV+) to the non-peptide-loaded CD4^+^ T-cells (HIV-) shown. (**e**) Overall killing capacity of CD8^+^ T-cell pool, to better reflect changes in frequency of HIV-specific CD8^+^ T-cells, was measured by normalising effector input as CD8^+^ T-cells (E) to peptide-loaded CD4^+^ T-cells (T) at various E:T. (**f**) Individual cytolytic capacity was measured by normalising effector input as tetramer^+^ HIV-specific CD8^+^ T-cell (E) to peptide-loaded CD4^+^ T-cells (T) at various effector(E):target(T) ratios. (n = 3; data shown as mean + SEM).

Supplementary Figure S5 **Phenotypic profile of pomalidomide-treated NK cells *ex vivo***. PBMC from PLHIV were treated with pomalidomide at 0.25 μM, or DMSO, for 72 hours *ex vivo*. (**a**) Pie charts represent the average percentage contribution to the total pool of PBMC. (**b**) The percentage of DNAM-1-expressing cells within the CD56^dim^CD16^+^ NK cell subset, and the TIGIT-expressing (TIGIT+) and TIGIT-negative (TIGIT-) CD56^dim^CD16^+^ NK cell subsets. MFC = Mean fold change. (n = 9; significance determined using the Wilcoxon matched-pairs signed rank test).
