# Supplementary figures and images for: Pomalidomide enhances CD8+ T and NK cell mediated killing of HIV-infected cells
Source: eBioMedicine. 2025 Nov 12;122:106004. doi: 10.1016/j.ebiom.2025.106004 (PMC12661362; doi:10.1016/j.ebiom.2025.106004)

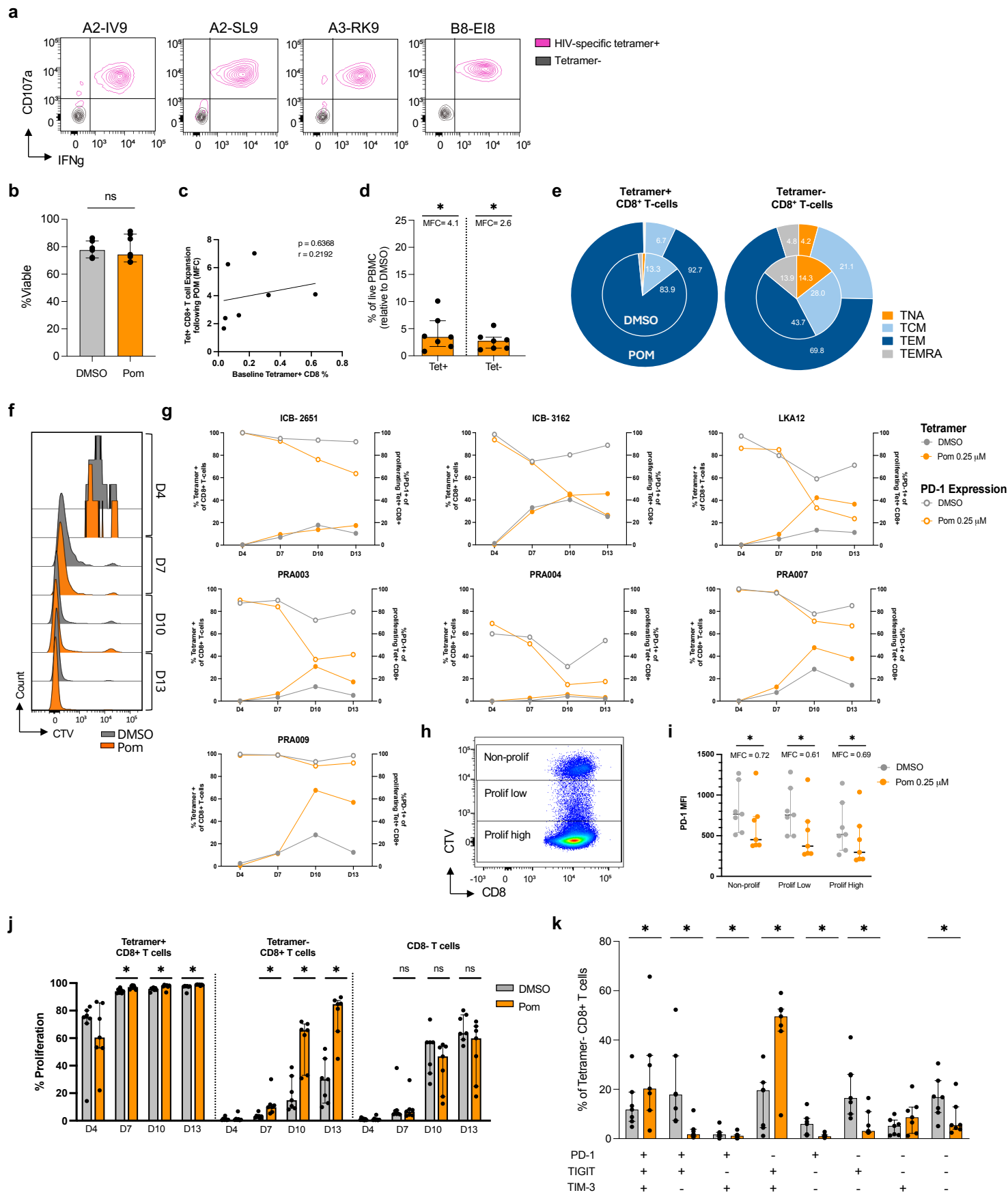

Supplement: Supplementary Figure S1 — Pomalidomide supports the proliferation of tetramer-negative CD8+ T-cells but to a lesser extent than tetramer+ HIV-specific CD8+ T-cells in the presence of HIV antigen. PBMC from PLHIV were pre-stained with the proliferation dye, CTV, and treated with DMSO or pomalidomide (0.25 μM) for 13 days, following exposure to an immunodominant HIV peptide. HIV-specific CD8+ T-cells were measured using a tetramer to the same immunodominant HIV peptide. (a) Validation of MHC-I:HIV tetramers, measuring degranulation (CD107a) and IFNγ in HIV-specific tetramer+ (pink) and tetramer- (grey) CD8+ T-cells (n = 4). (b) Frequency of viable PBMC after 13 days culture with pomalidomide (0.25 μM) or DMSO (n = 7). (c) Correlation between the baseline tetramer+ HIV-specific CD8+ T-cell frequency of total CD8+ T-cells and the pomalidomide-associated expansion of tetramer+ HIV-specific CD8+ T-cells (shown as fold change relative to DMSO). (d) Contribution of tetramer+ and tetramer- CD8+ T-cells to the live PBMC pool, shown as the fold change of pomalidomide-treatment relative to DMSO-treated conditions (e) Pie charts showing the median expression of CD8+ T-cell subsets within tetramer+ and tetramer- CD8+ T-cell populations. (f) Representative histogram of CTV division within tetramer+ HIV-specific CD8+ T-cells during 13-day culture with DMSO (grey) and pomalidomide (orange). (g) The frequency of tetramer-positive HIV-specific CD8+ T-cells (full circles, left y-axis), and the expression of PD-1 within the proliferating HIV-specific CD8+ T-cell population (circle outline, right y-axis) was quantified on days 4, 7, 10, and 13. DMSO indicated in grey, and pomalidomide-treated indicated in orange. Each plot represents a single donor tested. (h) Representative flow plot of the gating strategy for proliferation in DMSO and pomalidomide-treated HIV-specific CD8+ T-cells. (i) The MFI of PD-1 expression within the non-proliferating population, the lowly-, and highly-proliferative population, measured o [file mmc1.pdf]

**a**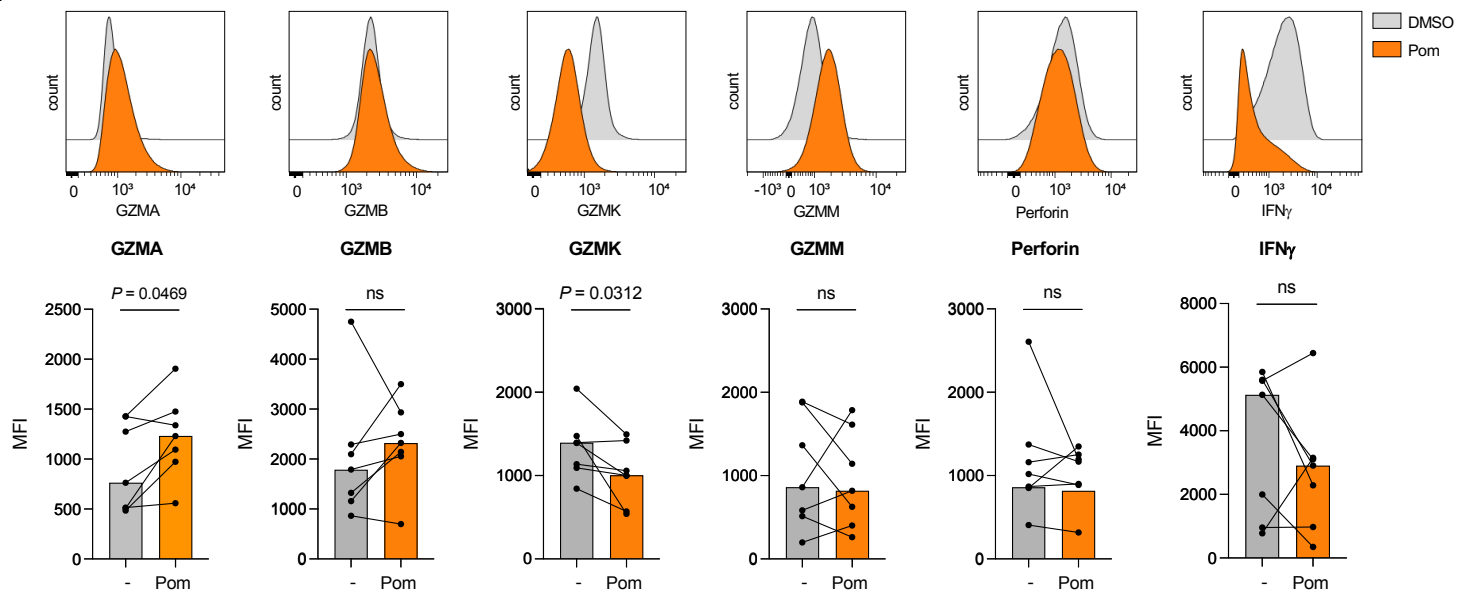**b**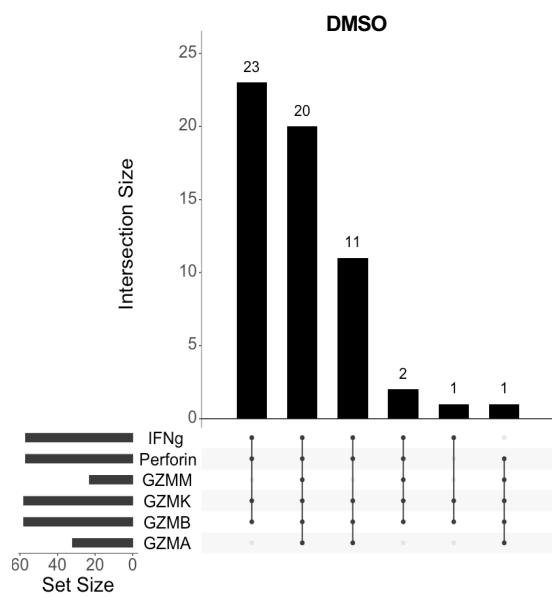**c**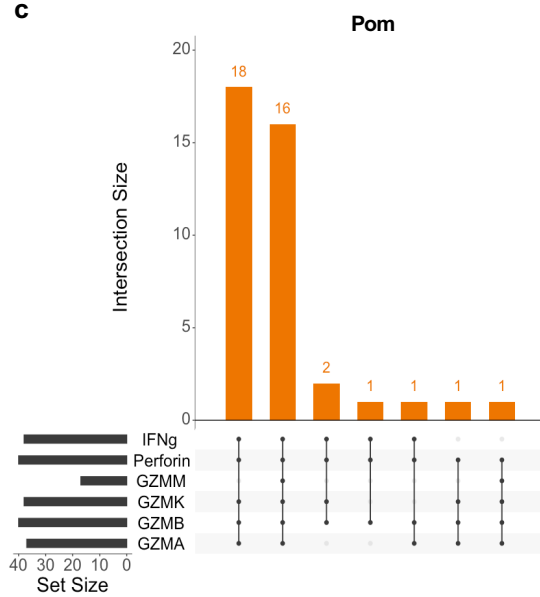

Supplement: Supplementary Figure S2 — Pomalidomide-associated enhanced proliferative capacity had no impact on the lytic capacity of HIV-specific CD8+ T-cells. PBMC from PLHIV were treated with DMSO or pomalidomide (0.25 μM) for 13 days, following exposure to an immunodominant HIV peptide. PBMC were then re-stimulated with the same HIV peptide to measure cytotoxic molecule production. (a) Representative histograms and median fluorescence index (MFI) expression of cytotoxic molecules and cytokines in degranulating (CD107a+) HIV-specific CD8+ T-cells. (b-c) Upset plots representing the major co-expression profiles of cytotoxic molecules within the degranulating fraction of (b) DMSO-treated and (c) pomalidomide-treated tetramer+ HIV-specific CD8+ T-cells. Intersection size reflects the frequency of the population co-expressing the indicated cytotoxic molecules listed below. Set size represents the expression frequency of the singular cytotoxic molecule. (n = 7; significance determined using Wilcoxon matched-pairs signed rank test; ns, not significant). [file mmc2.pdf]

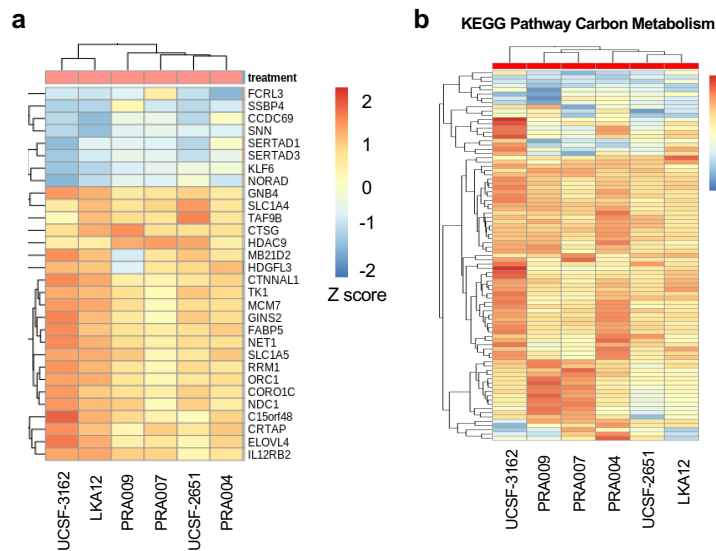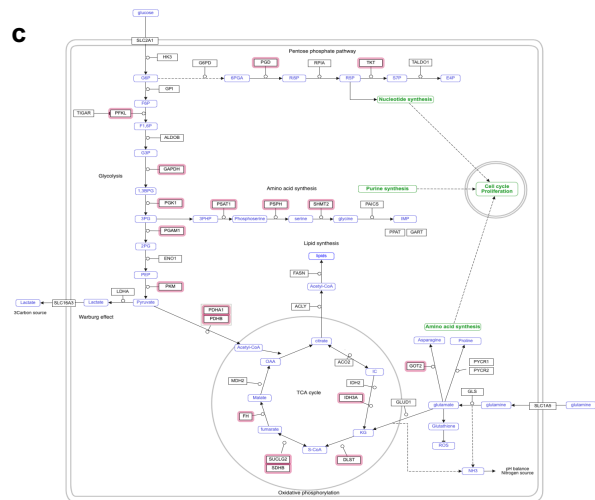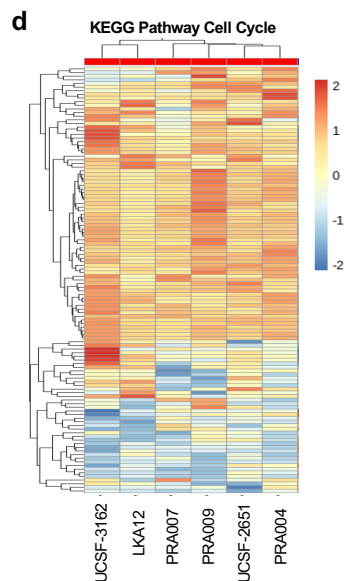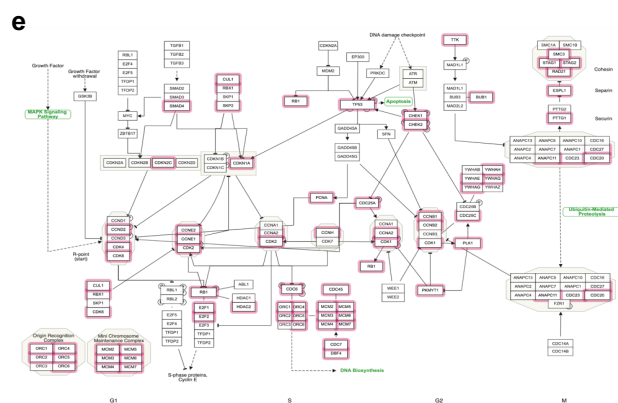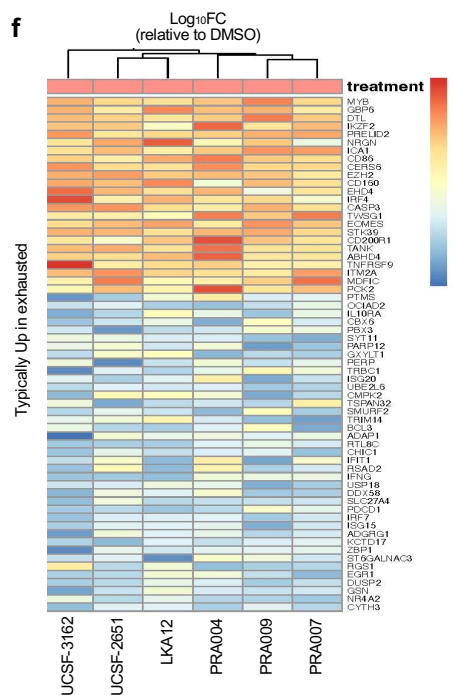

Supplement: Supplementary Figure S3 — Pomalidomide reduces the core exhaustion profile of HIV-specific CD8+ T-cells whilst promoting cell cycle progression. Tetramer+ HIV-specific CD8+ T-cells were sorted and sequenced using Illumina bulk RNA sequencing (n = 7). (a) Heatmap showing the top 30 significant differentially expressed genes (DEG) in pomalidomide-treated (0.25 μM) HIV-specific CD8+ T-cells relative to DMSO-treated HIV-specific CD8+ T-cells. Statistical significance determined as FDR<0.05. (b) Heatmap of the genes implicated in carbon metabolism in KEGG pathway analysis, with DEGs from pomalidomide-treated HIV-specific CD8+ T-cells, relative to DMSO, shown. (c) Protein interaction networks implicated in carbon metabolism. Analysis based on KEGG pathway results, performed using protein interactions generated and visualised using Cytoscape. Genes significantly upregulated by pomalidomide highlighted in red. (d) Heatmap of the genes implicated in cell cycle, according to KEGG pathway analysis, with DEGs from pomalidomide-treated HIV-specific CD8+ T-cells, relative to DMSO, shown. (e) Protein interaction networks implicated in cell cycle. Analysis based on KEGG pathway results, performed using protein interactions generated and visualised using Cytoscape. Genes significantly upregulated by pomalidomide highlighted in red. (f-g) Heatmaps of the significantly altered ‘core exhaustion’ genes in pomalidomide-treated HIV-specific CD8+ T-cells relative to DMSO. (F) Genes typically upregulated in exhaustion, based on the ‘core exhaustion profile’. (G) Genes typically downregulated in exhaustion, based on the ‘core exhaustion profile’. [file mmc3.pdf]

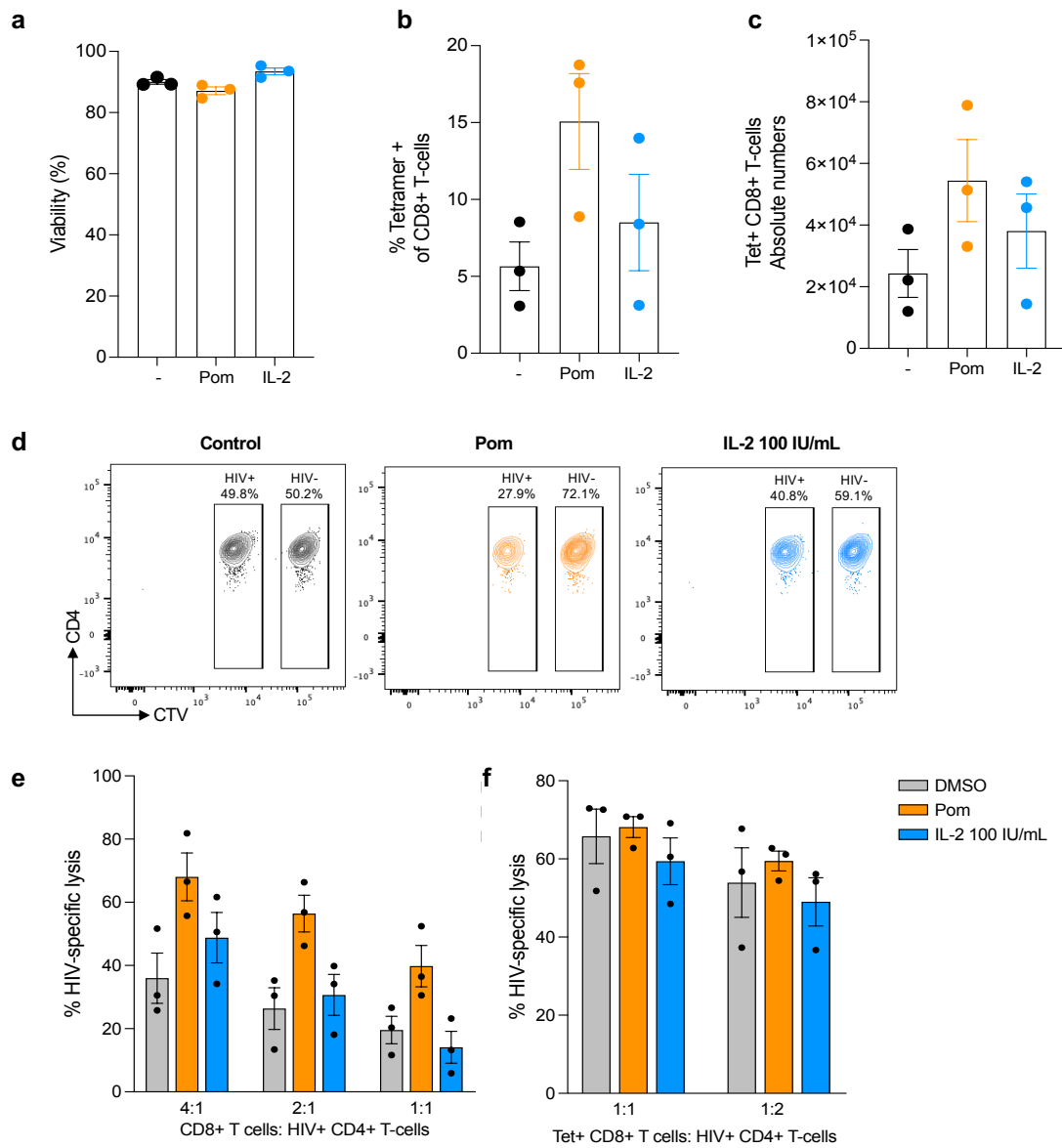

Supplement: Supplementary Figure S4 — Comparison of pomalidomide to high-dose IL-2 on the expansion and killing capacity of HIV-specific CD8+ T-cells. PBMC from PLHIV were treated with DMSO, pomalidomide (0.25 μM) or high-dose IL-2 (100 IU/mL) for 13 days, following exposure to an immunodominant HIV peptide. (a) Frequency of viable PBMC after 13 days culture with DMSO, pomalidomide or high-dose IL-2. (b) Frequency of tetramer+ HIV-specific CD8+ T-cells of CD8+ T-cell pool. (c) Absolute number of tetramer+ HIV-specific CD8+ T-cells. (d) Killing capacity was measured using the CD8+ T-cell killing assay. PBMC from PLHIV were stimulated with a HIV-immunodominant peptide and treated with DMSO or pomalidomide for 13 days. Purified CD8+ T-cells were co-cultured with autologous CD4+ T-cells stained with two CTV concentrations, with the lower concentration loaded with the HIV immunodominant peptide. HIV-specific lysis was calculated as the relative killing of the peptide-loaded CD4+ T-cells to the non-peptide-loaded CD4+ T-cells. Representative flow gating of the peptide-loaded CD4+ T-cells (HIV+) to the non-peptide-loaded CD4+ T-cells (HIV-) shown. (e) Overall killing capacity of CD8+ T-cell pool, to better reflect changes in frequency of HIV-specific CD8+ T-cells, was measured by normalising effector input as CD8+ T-cells (E) to peptide-loaded CD4+ T-cells (T) at various E:T. (f) Individual cytolytic capacity was measured by normalising effector input as tetramer+ HIV-specific CD8+ T-cell (E) to peptide-loaded CD4+ T-cells (T) at various effector(E):target(T) ratios. (n = 3; data shown as mean + SEM). [file mmc4.pdf]

**a**

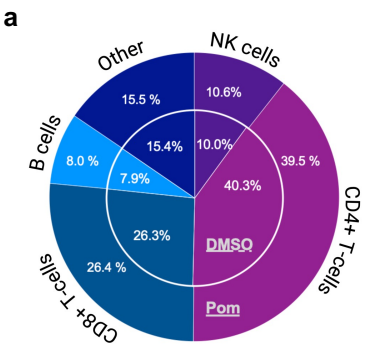

**b**

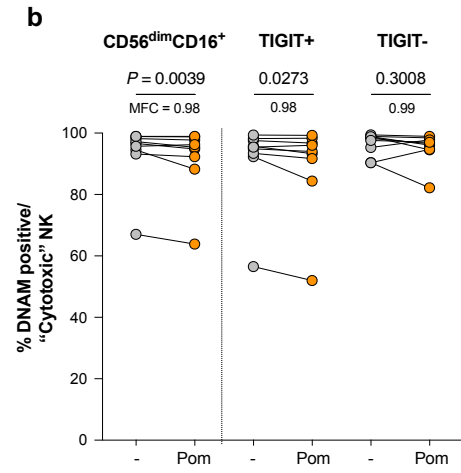

Supplement: Supplementary Figure S5 — Phenotypic profile of pomalidomide-treated NK cells ex vivo. PBMC from PLHIV were treated with pomalidomide at 0.25 μM, or DMSO, for 72 hours ex vivo. (a) Pie charts represent the average percentage contribution to the total pool of PBMC. (b) The percentage of DNAM-1-expressing cells within the CD56dimCD16+ NK cell subset, and the TIGIT-expressing (TIGIT+) and TIGIT-negative (TIGIT-) CD56dimCD16+ NK cell subsets. MFC = Mean fold change. (n = 9; significance determined using the Wilcoxon matched-pairs signed rank test). [file mmc5.pdf]
