# Supplementary material for: Pomalidomide enhances CD8+ T and NK cell mediated killing of HIV-infected cells
Source: eBioMedicine. 2025 Nov 12;122:106004. doi: 10.1016/j.ebiom.2025.106004 (PMC12661362; doi:10.1016/j.ebiom.2025.106004)
Supplement: Supplementary Tables [file mmc6.docx]

**Supplementary Results**

# **Supplementary Tables**

# **Table S1. Clinical characteristics of study participants living with HIV.**

| Participant ID | Age (years) | Sex | Race | HIV diagnosis | Years since HIV diagnosis | CD4^+^ count (cells/μL) | CD4^+^ (%) | CD8^+^ count (cells/μL) | CD8^+^ (%) | Nadir CD4^+^ count (cells/μL) | ART regimen | VL (copies/mL) | Peak VL (copies/mL) | Duration HIV RNA<50 copies (years) |
| --- | --- | --- | --- | --- | --- | --- | --- | --- | --- | --- | --- | --- | --- | --- |
| PRA001 | 64 | Male | Caucasian | 1985 | 31 | 403 | 24 | 1061 | 63 | 10 | ATV, TDF/FTC | <20 | 148,430 | 18 |
| PRA002 | 48 | Male | Caucasian | 2006 | 10 | 1460 | 47 | 793 | 26 | 698 | ABC/3TC, EFV | <20 | NA | 12 |
| PRA003 | 49 | Male | Caucasian | 1997 | 19 | 590 | 31 | 767 | 29 | 218 | TDF/FTC, DRV, RTV | <20 | 78,300 | 15 |
| PRA004 | 55 | Male | Caucasian | 1996 | 20 | 1036 | 40 | 1069 | 42 | 266 | TAF/FTC, DTG | <20 | 100,000 | 15 |
| PRA006 | 48 | Male | Caucasian | 2011 | 6 | 864 | 38 | 864 | 39 | 538 | EVG/TAF/FTC/COBI | <20 | 118,800 | 10 |
| PRA007 | 47 | Male | Caucasian | 2001 | 15 | 705 | 32 | 1034 | 47 | 122 | DRV/COBI, TAF/FTC | <20 | 548,000 | 10 |
| PRA008 | 38 | Male | Other (PNG) | 2006 | 11 | 281 | 25 | 328 | 30 | 168 | EVG/TAF/FTC/COBI | <20 | 63,300 | 13 |
| PRA009 | 49 | Male | Caucasian | 2010 | 9 | 474 | 25 | 1085 | 56 | 42 | EVG/TAF/FTC/COBI | <20 | 211,930 | 13 |
| UCSF 2651 | 52 | Male | Caucasian | 2001 | 17.4 | 655 | 37 | 681 | 39 | 275 | ABC/DTG/3TC | <40 | 45,069 | 14 |
| UCSF 3162 | 56 | Male | Caucasian | 1987 | 31.0 | 586 | 37 | 471 | 30 | 200 | DRV, RTV, ABC/DTG/3TC | <40 | 171,000 | 11.5 |
| LKA04 | 56 | Male | Caucasian | 1977 | 29.0 | 769 | 35 | 530 | 24 | 230 | ABC/3TC/DTG | <20 | NA | 28 |
| LKA12 | 57 | Male | Caucasian | 1996 | 20.0 | 744 | 37 | 932 | 46 | 624 | RAL, DRV | <20 | NA | 21 |
| LKA16 | 67 | Male | Caucasian | 2005 | 11.0 | 534 | 39 | 315 | 35 | 315 | ABC/3TC/DTG | <20 | NA | 10 |
| LKA18 | 60 | Male | Caucasian | 1993 | 25.0 | 312 | 19 | 312 | 34 | 2 | TAF/FTC | <20 | NA | 12 |
| LKA19 | 28 | Male | Caucasian | 2014 | 4.0 | 1072 | 42 | 857 | 33 | 731.9 | TAF/FTC/RPV | NA | NA | 4 |
| LAT21 | 62 | Male | Caucasian | 2012 | 10 | 742 | NA | NA | NA | 324 | 3TC/DTG | NA | NA | 6 |
| LAT22 | 57 | Male | Caucasian | 2010 | 12 | 889 | NA | NA | NA | 442 | BIC/FTC/TAF | NA | NA | 11 |
| Mean  (SD) | 52.5 (9.6) | NA | NA | NA | 16.5 (8.5) | 712.7 (297.6) | 33.9 (7.7) | 739.9 (297.6) | 38.2 (11.05) | 306.2 (229.0) | NA | NA | 164,981 (153,217) | 13.15  (5.5) |

ART: Antiretroviral therapy; VL: viral load; NA: not available. ABC,abacavir; ATV,atazanavir; COBI,cobicistat; DRV,darunavir; DTG,dolutegravir; EFV,efavirenz; EVG,elvitegravir; FTC,emtricitabine; RPV,rilpivirine; RTV,ritonavir; TAF,tenofoviralafenamide; 3TC,lamivudine; TDF,tenofovirdisoproxilfumarate.

# **Reagent Validation Statement**

All reagents used in this study, with the exception of the in-house generated HIV-specific tetramers, were commercially sourced. The vendor, catalogue numbers, Research Resource identifier (RRID), and validation reference for cell lines used in this study are found in Table S2. The antigen specificity, host and isotype, fluorophore conjugate, clone, working dilution, vendor, catalogue number, RRID, and validation reference for commercially sourced antibodies are found in Tables S3-S6.

# **Table S2. Cell lines**

| **Reagent or Resource** | **Vendor/Supplier** | **Cat. no#** | **RRID** | **Application Reference** |
| --- | --- | --- | --- | --- |
| K562 | A. Barrow, The University of Melbourne, Australia; ATCC | CCL-243 | RRID: CVCL_0004 | / |
| LAV/8E5 | NIH | ARP-095 | RRID: CVCL_3484 | ^1^ |
| HEK 293T | NIH | / | / | ^2^ |
| TZM-bl | NIH | HRP-8129 | RRID: CVCL-3484 | ^2^ |

# **Table S3. Spectral flow cytometry phenotyping antibody panel.**

| **Antigen** | **Host, Isotype** | **Conjugate** | **Clone** | **Dilution** | **Vendor** | **Cat#** | **RRID** |
| --- | --- | --- | --- | --- | --- | --- | --- |
| CD3 | Mouse, IgG1, κ | BV750 | SK7 | 1:100 | Biolegend | 344846 | AB_2800923 |
| CD4 | Mouse, IgG2b, κ | PE-Cy7 | OKT4 | 1:6400 | Biolegend | 317414 | AB_571959 |
| CD8 | Mouse, IgG1, κ | PerCP-Cy5.5 | SK1 | 1:100 | Biolegend | 344710 | AB_2044010 |
| CD45RA | Mouse, IgG2b, κ | AF488 | HI100 | 1:100 | Biolegend | 304114 | AB_528816 |
| CD197 (CCR7) | Mouse, IgG2a, κ | AF700 | G043H7 | 1:200 | Biolegend | 353244 | AB_2617001 |
| CD27 | Mouse, IgG1, κ | BV785 | O323 | 1:100 | Biolegend | 302832 | AB_2562674 |
| CD25 | Mouse, IgG1, κ | APC-Fire750 | BC96 | 1:100 | Biolegend | 302642 | AB_2632839 |
| HLA-DR | Mouse, IgG2a, κ | BV570 | L243 | 1:200 | Biolegend | 307638 | AB_2650882 |
| CD38 | Mouse, IgG1, κ | BV510 | HIT2 | 1:50 | Biolegend | 303540 | AB_2616792 |
| CD279 (PD-1) | Mouse, IgG1, κ | BUV661 | EH12.1 | 1:50 | BD Biosciences | 750260 | AB_2874457 |
| TIGIT | Mouse, IgG2a, κ | BV605 | A15153G | 1:50 | Biolegend | 372712 | AB_2632927 |
| CD366 (TIM-3) | Mouse, IgG1, κ | Superbright436 | F38-2E2 | 1:100 | Invitrogen | 62-3109-42 | AB_2637363 |
| CD274 (PD-L1) | Mouse, IgG1, κ | BUV496 | MIH1 | 1:100 | BD Biosciences | 741189 | AB_2870754 |
| CD57 | Mouse, IgM | eFluor450 | TB01 | 1:200 | Invitrogen | 48-0577-42 | AB_2016680 |
| CD56 | Mouse, IgG1, κ | PerCP-eFluor710 | TULY56 | 1:200 | Invitrogen | 46-0566-42 | AB_2637487 |
| CD16 | Mouse, IgG1, κ | PE/Dazzle594 | 3G8 | 1:500 | Biolegend | 302054 | AB_2563639 |
| CD159a (NKG2A) | Recombinant human IgG1 | APC | REA110 | 1:400 | Miltenyi-Biotec | 130-113-563 | AB_2726170 |
| CD159c (NKG2C) | Mouse, IgG1, κ | BV480 | 134591 | 1:100 | BD Biosciences | 748168 | AB_2872629 |
| CD314 (NKG2D) | Mouse, IgG1, κ | BB515 | 1DII | 1:25 | BD Biosciences | 564566 | AB_2738848 |
| CD337 (NKp30) | Mouse, IgG1, κ | BUV805 | p30-15 | 1:50 | BD Biosciences | 749127 | AB_2873516 |
| CD336 (NKp44) | Mouse, IgG1, κ | BUV395 | p44-8 | 1:50 | BD Biosciences | 744305 | AB_2742135 |
| CD226 (DNAM-1) | Mouse, IgG1, κ | BUV563 | DX11 | 1:50 | BD Biosciences | 748429 | AB_2872848 |
| CD14 | Mouse, IgG2b, κ | BV421 | Mϕp9 | 1:200 | BD Biosciences | 563743 | AB_2744289 |
| CD40 | Mouse, IgG1, κ | BV711 | 5C3 | 1:50 | Biolegend | 334334 | AB_2564212 |
| CD86 | Mouse, IgG2b, κ | AF647 | IT2.2 | 1:200 | Biolegend | 305416 | AB_528883 |
| CD123 | Mouse, IgG1, κ | PE/Cy5 | 6H6 | 1:500 | Biolegend | 306008 | AB_493574 |
| CD11c | Mouse, IgG1, κ | BUV737 | B-ly6 | 1:200 | BD Biosciences | 741827 | AB_2871162 |
| CD155 | Mouse, IgG1, κ | PE | SKII.4 | 1:400 | BD Biosciences | 566718 | AB_2739771 |
| CD19 | Mouse, IgG1, κ | BV650 | HIB19 | 1:50 | Biolegend | 302238 | AB_2562097 |

# **Table S4. HIV-specific CD8+ T-cell proliferation antibody panel.**

| **Antigen** | **Host, Isotype** | **Conjugate** | **Clone** | **Dilution** | **Vendor** | **Cat#** | **RRID** |
| --- | --- | --- | --- | --- | --- | --- | --- |
| CD3 | Mouse, IgG2a, κ | AF700 | HIT3a | 1:100 | Biolegend | 300324 | AB_493739 |
| CD8a | Mouse, IgG1, κ | BUV805 | SK1 | 1:100 | BD Biosciences | 612889 | AB_2044010 |
| CD45RA | Mouse, IgG2b, κ | PerCP Cy5.5 | HI100 | 1:200 | Biolegend | 304122 | AB_893357 |
| CD279 (PD-1) | Mouse, IgG1, κ | BUV661 | EH12.1 | 1:50 | BD Biosciences | 750260 | AB_2874457 |
| CD27 | Mouse, IgG1 | BV711 | L128 | 1:100 | BD Biosciences | 563167 | AB_2738042 |

# **Table S5. CD8+ T-cell intracellular antibody panel.**

| **Antigen** | **Host, Isotype** | **Conjugate** | **Clone** | **Dilution** | **Vendor** | **Cat#** | **RRID** |
| --- | --- | --- | --- | --- | --- | --- | --- |
| **Surface staining** | | | | | | | |
| CD107a | Mouse, IgG1, κ | BUV395 | H4A3 | 1:100 | BD Biosciences | 565113 | AB_2739073 |
| CD3 | Mouse, IgG2a, κ | BV605 | OKT3 | 1:50 | Biolegend | 317322 | AB_2561911 |
| CD8a | Mouse, IgG1, κ | BUV805 | SK1 | 1:100 | BD Biosciences | 612889 | AB_2044010 |
| CD279 (PD-1) | Mouse, IgG1, κ | BUV661 | EH12.1 | 1:50 | BD Biosciences | 750260 | AB_2874457 |
| **Intracellular Staining** | | | | | | | |
| Granzyme A | Mouse, IgG1, κ | AF488 | CB9 | 1:50 | Biolegend | 507212 | AB_528909 |
| Granzyme B | Mouse, IgG1, κ | AF700 | GB11 | 1:50 | BD Biosciences | 560213 | AB_1645453 |
| Granzyme K | Mouse, IgG2a, κ | PerCP-eFluor710 | G3H69 | 1:33 | Invitrogen | 46-8897-42 | AB_2573854 |
| Granzyme M | Mouse, IgG1, κ | AF647 | 4B2G4 | 1:33 | BD Biosciences | 566996 | AB_2869996 |
| Perforin | Mouse, IgG1 | PE-Cy7 | B-D48 | 1:50 | Biolegend | 353316 | AB_2571973 |
| IFNγ | Mouse, IgG1, κ | BV785 | 4S.B3 | 1:25 | Biolegend | 502542 | AB_2563882 |

# **Table S6. NK cell Intracellular antibody panel.**

| **Antigen** | **Host, Isotype** | **Conjugate** | **Clone** | **Dilution** | **Vendor** | **Cat#** | **RRID** |
| --- | --- | --- | --- | --- | --- | --- | --- |
| **Surface staining** | | | | | | | |
| CD107a | Mouse, IgG1, κ | AF647 | H4A3 | 1:400 | BD Biosciences | 562622 | AB_2737684 |
| CD3 | Mouse, IgG1, κ | BV750 | SK7 | 1:100 | Biolegend | 344846 | AB_2800923 |
| CD4 | Mouse, IgG2b, κ | PE-Cy7 | OKT4 | 1:6400 | Biolegend | 317414 | AB_571959 |
| CD8a | Mouse, IgG1, κ | BUV805 | SK1 | 1:100 | BD Biosciences | 612889 | AB_2044010 |
| CD14 | Mouse, IgG2b, κ | BV421 | Mϕp9 | 1:200 | BD Biosciences | 563743 | AB_2744289 |
| CD56 | Mouse, IgG1, κ | PerCP-eFluor710 | TULY56 | 1:200 | Invitrogen | 46-0566-42 | AB_2637487 |
| CD16 | Mouse, IgG1, κ | PE/Dazzle594 | 3G8 | 1:800 | Biolegend | 302054 | AB_2563639 |
| CD19 | Mouse, IgG1, κ | BV650 | HIB19 | 1:50 | Biolegend | 302238 | AB_2562097 |
| **Intracellular Staining** | | | | | | | |
| IFNγ | Mouse, IgG1, κ | BUV395 | B27 | 1:100 | BD Biosciences | 563563 | AB_2738277 |
| Granzyme B | Mouse, IgG1, κ | FITC | GB11 | 1:50 | BD Biosciences | 560211 | AB_1645488 |
| TNFα | Mouse, IgG1, κ | AF700 | Mab11 | 1:166 | BD Biosciences | 557996 | AB_396978 |
| IL-2 | Rat, IgG2a, κ | PE | MQI-17H12 | 1:10 | BD Biosciences | 559334 | AB_397231 |
